# Network integration and modelling of dynamic drug responses at multi-omics levels

**DOI:** 10.1038/s42003-020-01302-8

**Published:** 2020-10-15

**Authors:** Nathalie Selevsek, Florian Caiment, Ramona Nudischer, Hans Gmuender, Irina Agarkova, Francis L. Atkinson, Ivo Bachmann, Vanessa Baier, Gal Barel, Chris Bauer, Stefan Boerno, Nicolas Bosc, Olivia Clayton, Henrik Cordes, Sally Deeb, Stefano Gotta, Patrick Guye, Anne Hersey, Fiona M. I. Hunter, Laura Kunz, Alex Lewalle, Matthias Lienhard, Jort Merken, Jasmine Minguet, Bernardo Oliveira, Carla Pluess, Ugis Sarkans, Yannick Schrooders, Johannes Schuchhardt, Ines Smit, Christoph Thiel, Bernd Timmermann, Marcha Verheijen, Timo Wittenberger, Witold Wolski, Alexandra Zerck, Stephane Heymans, Lars Kuepfer, Adrian Roth, Ralph Schlapbach, Steven Niederer, Ralf Herwig, Jos Kleinjans

**Affiliations:** 1 Functional Genomics Center, ETH Zurich, Switzerland; 2 Department of Toxicogenomics, Maastricht University, Maastricht, The Netherlands; 3 Roche Pharma Research and Early Development, Roche Innovation Center Basel, Basel, Switzerland; 4 Genedata AG, Basel, Switzerland; 5 Insphero AG, Schlieren, Switzerland; 6 European Molecular Biology Laboratory, European Bioinformatics Institute (EMBL-EBI), Hinxton, UK; 7 MicroDiscovery GmbH, Berlin, Germany; 8 Institute of Applied Microbiology, RWTH, Aachen, Germany; 9 Department of Computational Molecular Biology, Max-Planck-Institute for Molecular Genetics, Berlin, Germany; 10 Max-Planck-Institute for Molecular Genetics, Sequencing Unit, Berlin, Germany; 11 Department of Biomedical Engineering, King’s College London, London, UK; 12 CARIM School for Cardiovascular Diseases, Maastricht University, Maastricht, The Netherlands

**Keywords:** Protein-protein interaction networks, Biochemical networks, Data integration

## Abstract

Uncovering cellular responses from heterogeneous genomic data is crucial for molecular medicine in particular for drug safety. This can be realized by integrating the molecular activities in networks of interacting proteins. As proof-of-concept we challenge network modeling with time-resolved proteome, transcriptome and methylome measurements in iPSC-derived human 3D cardiac microtissues to elucidate adverse mechanisms of anthracycline cardiotoxicity measured with four different drugs (doxorubicin, epirubicin, idarubicin and daunorubicin). Dynamic molecular analysis at in vivo drug exposure levels reveal a network of 175 disease-associated proteins and identify common modules of anthracycline cardiotoxicity in vitro, related to mitochondrial and sarcomere function as well as remodeling of extracellular matrix. These in vitro-identified modules are transferable and are evaluated with biopsies of cardiomyopathy patients. This to our knowledge most comprehensive study on anthracycline cardiotoxicity demonstrates a reproducible workflow for molecular medicine and serves as a template for detecting adverse drug responses from complex omics data.

## Abstract (web summary)

Using a network propagation approach with integrated multi-omic data, Selevsek et al. develop a reproducible workflow for identifying drug toxicity effects in cellular systems. This is demonstrated with the analysis of anthracycline cardiotoxicity in cardiac microtissues under the effect of multiple drugs.

## Introduction

Personalized medicine targets an individual’s pathology whilst simultaneously aiming to minimize therapy side effects introduced by drug toxicities. Crucial for realizing this goal is a comprehensive characterization of adverse pathways across multiple regulatory systems. In this context the identification of mechanisms through application of multiple-omics technologies that enable capturing the full width of molecular responses upon drug treatment combined with integrative data analysis approaches has been defined as the key strategy for solving the task^1^.

Such integrated approaches have impact on standard-of-care therapies as well as on the development of novel drugs. In particular, adverse effects of anti-cancer drugs on the heart are a growing clinical problem with the ever increasing number of cancer patients^2^. Moreover, unforeseen cardiovascular toxicity is among the most important reason for drug candidate failure^3,4^ and accounts for 14% of drug withdrawals upon market introduction due to adverse reactions^5–8^. This is mainly due to preclinical non-human test models for drug toxicity that translate only poorly to the human conditions; in fact, across a range of pharmaceuticals it has been demonstrated that to the best, only 63% of compounds show concordance of toxicity between animal test results and human responses^9,10^. Overall, an understanding of underlying mechanisms of toxicity has been recognized as one of the most effective ways to improve drug safety. Against this background, Collins et al.^11^ proposed to bypass animal-based drug toxicity testing, by developing molecular response patterns generated from human cell-based in vitro systems that are indicative of human disease phenotypes, and by identifying pivotal signaling pathways leading to toxicity. Here, the toxicogenomics approach has been claimed to be capable of identifying new genomic entities yielding innovative prediction models for adverse drug reactions thereby improving risk assessment^12^. An exemplar study already demonstrated the usefulness of global gene expression-based in vitro modeling for predicting drug-induced hepatic cytotoxicity^13^. But a true integrative understanding of molecular mechanisms of adverse drug reactions can only be accomplished by interrogating different levels of molecular activities in a dynamic fashion, and this approach requires (i) capturing of the dynamic responses across time and dose in the system under study enabling dynamic network inference and quantitative modeling^14,15^, (ii) identification of mechanistic networks of interacting proteins that are responsible for the systemic drug response^16,17^, and (iii) validation of these networks in human patients.

Consequently, in a yet unmet endeavor, we obtained proof-of-principle for such an integrated approach with respect to cardiovascular drug toxicity. We generated dose-over-time cross-omics data from an advanced iPSC-derived human cardiac 3D microtissue model exposed to physiologically relevant doses of anthracyclines (ACs) over a time period of 14 days^18^. ACs are widely used chemotherapies despite the fact that they induce cardiotoxicities in up to 23% of the patients. AC-induced cardiotoxicity (ACT) represents a cumulative systemic effect over time and refers to changes in myocardial functions for example in left-ventricular ejection fraction (LVEF), diastolic functions, arrhythmias as well as cardiac stress responses. The major molecular hallmarks of ACT include the generation of reactive oxygen species (ROS) and changes in mitochondrial response pathways that ultimately lead to mitochondrial damage, the interference of ACs with topoisomerase 2 (*TOP2B*) and thus the disruption of gene regulation and DNA damage repair mechanisms as well as changes in sarcomere function^19,20^.

We have generated dynamic quantitative proteomics (LC–MS), transcriptomics (RNA-seq) and methylation (MeDIP-seq) landscapes from the cardiac cell model for four widely used anthracyclines (doxorubicin (DOX), epirubicin (EPI), idarubicin (IDA), and daunorubicin (DAU)) and conducted an integrated computational approach using the results of longitudinal expression analyses and network propagation based on insulated heat diffusion^21^. We identified a network of 175 proteins representing the common signature of ACT. We then undertook the challenge of acquiring cardiac biopsies taken from patients with and without historic AC treatment and now suffering from chronic cardiac failure and in a yet untried approach, compared the in vitro results with proteome analysis of these target tissue samples. We demonstrate that proteins of the ACT network were (i) clinically transferable to patients suffering from drug-induced cardiomyopathies and (ii) physiologically relevant as tested with a previously defined model for predicting ACT of the cardiomyocyte mitochondrion^22^ and thus that network-based data integration has major potential for advancing the field of molecular medicine.

## Results

### Experimental design

A workflow of the analyses is shown in Supplementary Fig. 1. We have challenged an iPSC-derived human 3D cardiac microtissue cell model (see “Methods” section) over a 14 days period with four anthracycline drugs (DOX; EPI; IDA; DAU) that were dissolved in DMSO at two physiologically relevant doses (therapeutic and toxic dose; Supplementary Table 1) as calculated by means of reversed pharmacokinetic modeling (Supplementary Fig. 2). We measured methylome, transcriptome, and proteome responses at both doses at seven time points over a 14 days period (2, 8, 24, 72, 168, 240, 336 h) using three replicate measurements per time point. In order to identify dynamically altered proteins and transcripts we compared the AC treatment time profiles with control profiles derived from time-matched DMSO-treated microtissues. The dynamic changes in proteome and transcriptome (372 different experiments in total; Supplementary Table 2) caused by the different AC treatments were integrated in a large protein–protein interaction network (PPI), and network propagation modeling was used to identify an ACT response network that reflects the major changes of the interactome with respect to AC treatment (Supplementary Methods). The ACT response network was subsequently evaluated in the context of cardiomyopathy patients as well as with an established computational model of the human cardiac mitochondrion.

### Methylation changes induced by ACs in 3D cardiac microtissues impact transcriptional regulation and gene expression

**Fig. 1:**
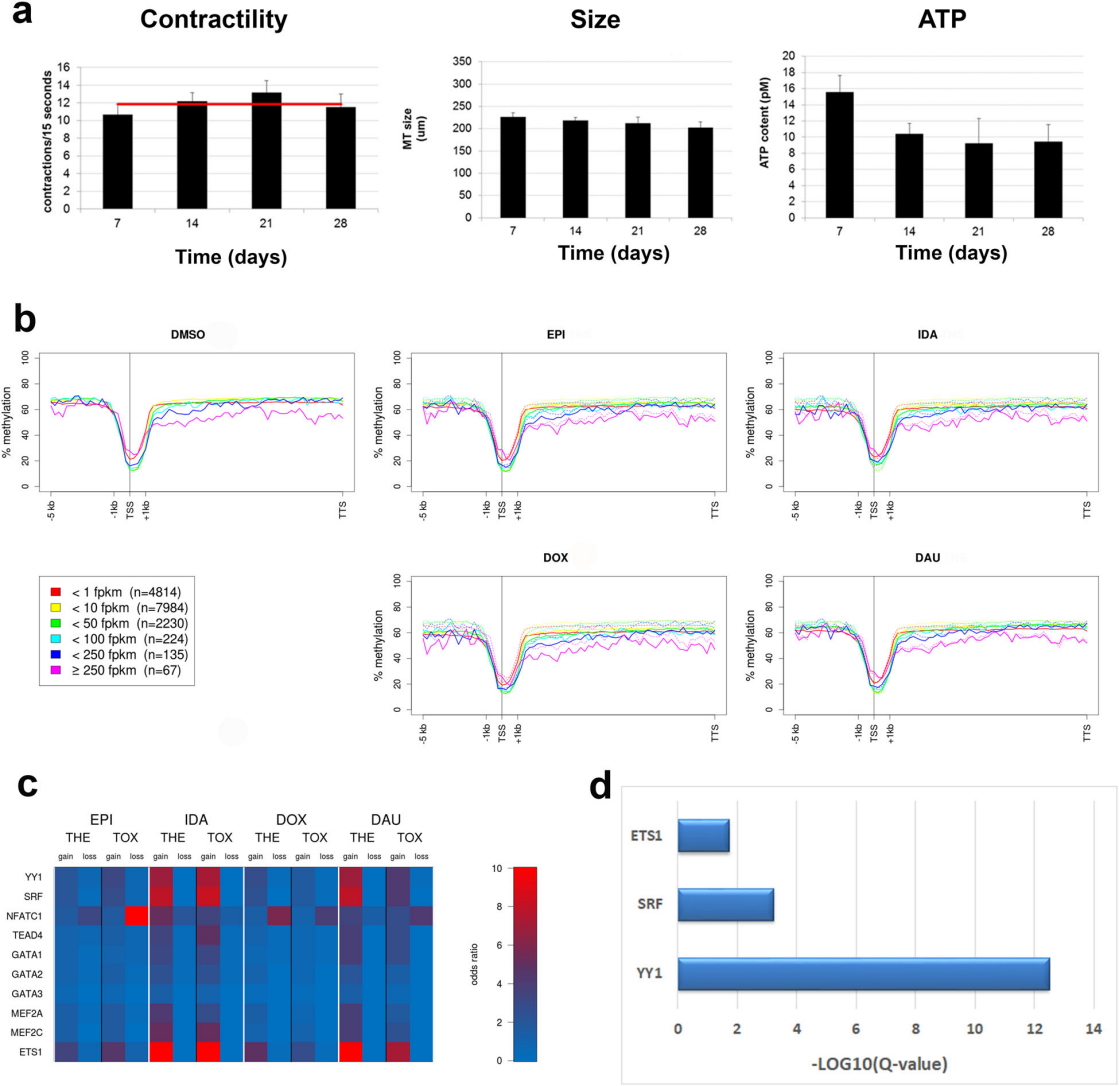
3D cardiac microtissues and genome-wide methylation. **a** Essential microtissue characteristics remain stable across a time period of 28 days. Contractions per 15 s, microtissue sizes and ATP content. Bars indicate measurements per week. Red line is the average over all 4 weeks. **b** Inverse correlation of gene body methylation and expression. Genes were classified into six classes of expression strengths based on the median fragments per kilobase of transcript per million reads (FPKM) over all time points and treatments. Methylation values (*Y*-axes) were averaged for each class of genes according to the following procedure: promoters and gene bodies were binned in 75 windows, 20 bins for the promoter (−5 kb), 1 bin for the transcription start site (TSS), 4 bins for the starting exons/introns (+1 kb) and 50 bins for the rest of the gene body (TTS: transcription termination site); for each gene % methylation in each bin was averaged over all seven time points for the specific treatment and then % methylation was averaged over all genes of the respective expression strength classes. In the plots of the AC-treated experiments the respective curves for the control experiments (DMSO) are shown as dotted lines. *X*-axes show the prototypical gene structure. TSS: transcription start site, TTS: transcription termination site. **c** Enrichment of TFBSs of cardiac transcription factors in DMRs that fall into gene promoters. Odds ratios (red = higher than expected) represent the ratio of the observed number of TFBSs that fall into DMRs versus the expected number. **d** Over-representation of transcription factor target sets in the list of 641 dynamic response proteins (next section). *X*-axis represents the strength of enrichment, i.e. –log10 of the over-representation *Q*-value.

Cardiac microtissues have been demonstrated to retain essential contractile properties of the heart and viability for up to 4 weeks and thus are suitable in vitro models for studying time-resolved drug toxicity mechanisms^23,24^. Microtissues are composed of iPSC-derived human cardiomyocyte and cardiac fibroblast cells (proportion 4:1). They show spontaneous contractile activity, Ca^2+^ responses, homogenous tissue without central necrosis, a spherical shape and were filled with myofibrils, a tissue structure characteristic for the mammalian heart. Essential functions such as contractility, microtissue size, and ATP generation and consumption are observable over the entire time period (Fig. 1a).

Methylation patterns are key characteristics of cellular identity. Genome-wide methylation profiles were generated with MeDIP-seq, and the enrichment signals were quantified as % methylation with a Bayesian approach using the QSEA tool^25^ (see “Methods” section). We examined whether the microtissues express previously found in vivo epigenetic characteristics of adult cardiomyocytes. In mouse as well as in human adult cardiomyocytes it has been observed that gene body methylation is inversely correlated with gene expression^26,27^. This overall trend in vivo indeed appears to be preserved in the iPSC-derived cardiac microtissues: Lowly expressed genes show a higher level of promoter and gene body methylation than highly expressed genes (Fig. 1b). Additionally, tissue-specific methylation has been charted previously among 30 human cell lines using whole-genome bisulfite sequencing^28^. We have investigated the heart-specific “dynamic” differentially methylated regions (DMRs) and found good agreement of methylation levels between 3D cardiac microtissues and human heart-specific cell lines (Supplementary Fig. 3). Thus, the cellular identity of iPSC-derived cardiac microtissues makes them a suitable model for the human heart muscle.

We applied pooled time point analysis (Supplementary Methods) with the longitudinal data to identify DMRs between treated and control microtissues for each drug and dose using QSEA^25^. Most DMRs are observable in intergenic (42–52%) and intronic (41–43%) regions with only a small fraction (5–8%) in promoter regions (Supplementary Figs. 4 and 5). In total, 2145 and 776 DMRs (*q*-value *Q* < 0.01) were found consistently differentially methylated at therapeutic and toxic doses, respectively, with all four ACs. At therapeutic dose 984 DMRs were located in gene bodies (introns: 904, exons: 80) and 151 DMRs were found in promoter sequences, corresponding to 758 different genes. At toxic dose 322 DMRs were located in gene bodies (introns: 312, exons: 10) and 30 DMRs in promoter sequences, corresponding to 250 different genes. These genes enrich important pathways such as *adrenergic signaling in cardiomyocytes* (Supplementary Data 1), which has been reported to be compromised in iPSC-derived cardiomyocytes of patients with dilated cardiomyopathy (DCM)^29^.

To examine the association of AC-induced methylation patterns with transcriptional regulation, we contrasted DMRs against transcription factor-binding sites (TFBSs) as measured by ENCODE^30^. Enrichment of the number of TFBSs that overlap with DMRs compared to the total number of TFBSs was observed for transcription factors that are important in cardiac development, function and pathology^31,32^ (Fig. 1c). In particular, *YY1* (Ying Yang 1; odds ratios range between 2.67 and 7.74 across the different treatments and doses), *ETS1* (ETS Proto-Oncogene 1, transcription factor; 2.11–13.18) and *SRF* (serum response factor; 1.87–8.56) binding sites showed strong enrichment of TFBSs in hypermethylated DMRs, which indicates that gene regulation of these factors is impaired by AC-induced methylation demonstrating profound disturbance of regulatory pathways crucial for cardiac physiology (cf. “Discussion” section).

### Dynamic changes in the proteome and transcriptome reveal consequences of AC treatment for sarcomere and mitochondrial functions

For each treatment and dose time-resolved quantitative LC–MS analysis was performed and analyzed with longitudinal data analysis (Supplementary Methods) using a polynomial regression model (degree ≤ 2)^33^. This yielded for each protein an estimation of its temporal response as well as the significance of the deviation of the treatment temporal profile from the respective control profile (Supplementary Fig. 6; see “Methods” section). A total of 641 dynamic response proteins showed significant changes of time profiles with the different treatments and doses compared to DMSO control experiments (Supplementary Data 2). These proteins were enriched in the target sets of cardiac transcription factors *YY1*, *SRF,* and *ETS1* identified from methylation analysis in the previous section (Fig. 1d).

**Fig. 2:**
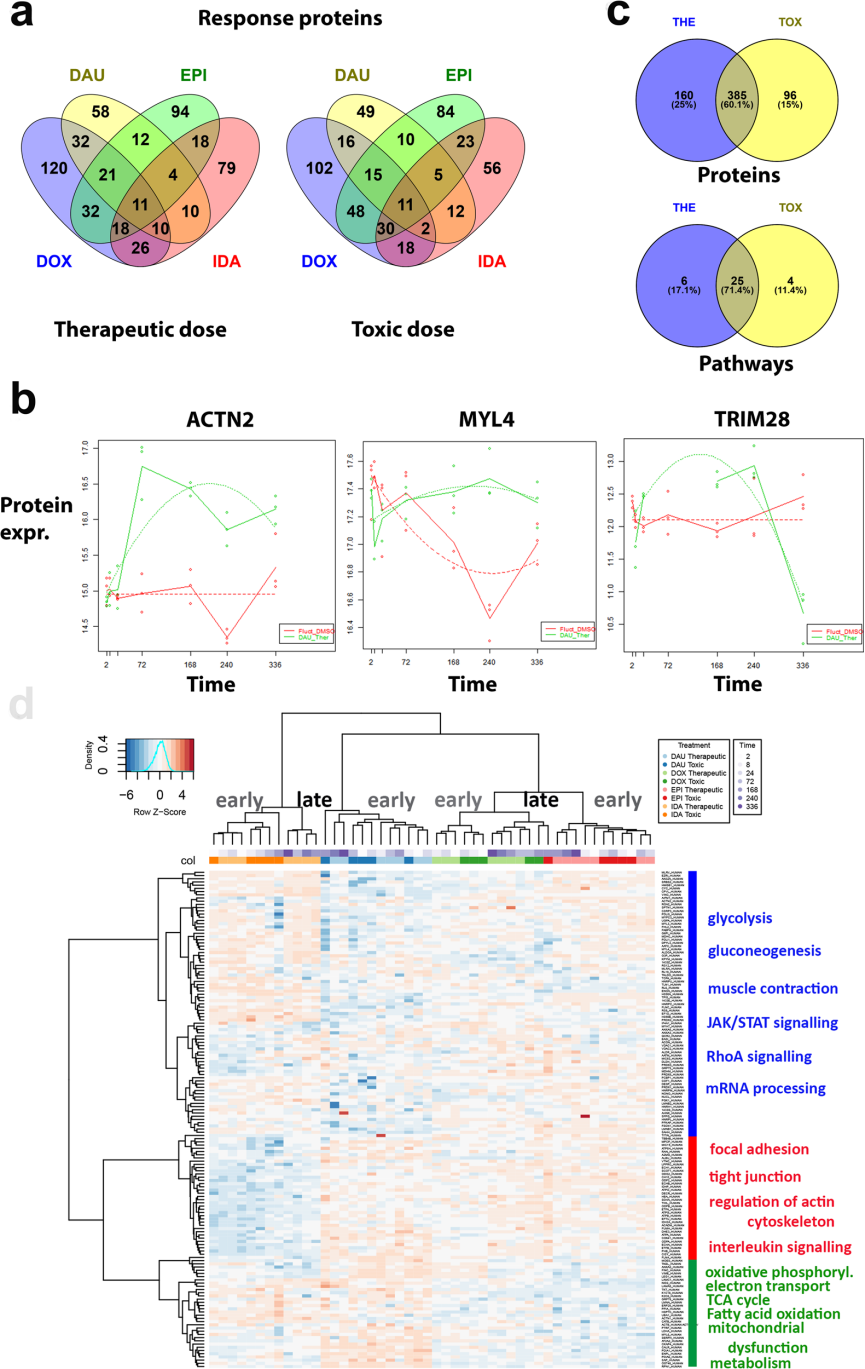
Dynamic proteome changes upon AC treatment. **a** VENN diagrams (generated with Venny 2.1.0) of dynamically altered proteins after AC treatment (DOX: blue, DAU: yellow, EPI: green, IDA: red) measured with LC–MS at therapeutic and toxic doses. **b** Examples of polynomial regression fit (degree ≤ 2) for significantly altered sarcomere genes (*ACTN2*, *MYL4*) and transcriptional regulators (*TRIM28*). The curves of the regression fit are plotted as dotted lines, solid lines connect the median values of the three replicates per time point to visualize the trend over time. Dots: experimental values, red curves: DMSO control experiments, green curves: AC treatment (DAU). *Y*-axes: normalized protein expression, *X*-axes: time. **c** VENN diagram showing overlap of proteins dynamically altered upon AC treatment according to therapeutic (THE) and toxic (TOX) doses. Overlap is counted with respect to proteins and enriched pathways. **d** Hierarchical clustering of protein expression over all experiments using the h.clust() method in R with Eucledian distance as distance measure and Ward’s method (option “ward.d2”) as agglomeration method. Upper panels code for the different treatments and the different time points per treatment. Right panel describes the major enriched functional pathways of the three main clusters of proteins.

ACs induced heterogeneous dynamic responses in terms of protein content with DOX (359 proteins at both doses) and EPI (313) affecting higher numbers of proteins followed by IDA (230) and DAU (200; Fig. 2a). Nonetheless enriched biological functions and pathways are shared by all ACs, in particular those related to sarcomere and mitochondrial function (Fig. 2b; Supplementary Data 2). Prominent diagnostic markers and related proteins of contractile units of cardiomyocytes were identified in the AC-challenged microtissues, for example cardiac muscle troponin T (*TNNT2*), which is dynamically altered with respect to EPI, IDA, and DAU at therapeutic doses and with DOX, EPI, and DAU at toxic doses. Time-point specific analysis (Supplementary Methods) comparing differential protein and gene expression of treated and control conditions shows up-regulation of *TNNT2* for most time points except for DAU (Supplementary Fig. 7). Other sarcomere-related proteins include further troponins (*TNNI1*, *TNNC2*), myosins (*MYBPC3*, *MYH4*, *MYH7*, *MYL2*, *MYL3*, *MYL4*, *MYL7*), tropomyosins (*TPM1*, *TPM2*, *TPM4*) among others. In total, 47 out of 199 sarcomere-related proteins (GO:0030017; enrichment *q*-value *Q* = 1.28E−25) showed a significantly altered time profile upon AC treatment when compared to control microtissues. Further largely enriched functional groups relate to mitochondrial dysfunction and response pathways^34^ in particular electron transport chain (GO:0022900; 39 out of 180 proteins, *Q* = 1.30E–19), respiratory chain complex (GO:0098803; 20 out of 85, *Q* = 3.04E–11) and response to oxidative stress (GO:0006979; 44 out of 426, *Q* = 2.85E−10).

Dynamic AC responses at the proteome level were fairly similar with respect to both dosages. 545 and 481 proteins were found significant at therapeutic and toxic doses, respectively, with 385 proteins in common (Jaccard agreement 0.60; Fig. 2c). On the level of pathways, similarity is even higher (Jaccard agreement 0.71) with a slight overall increase of pathway enrichment with toxic compared to therapeutic dose. Clustering of protein expression over all experiments shows that with every AC time points are very well discriminated into earlier (2–72 h) and later responses (168–336 h). Furthermore, groups of proteins can be separated into functional hallmarks of cardiotoxicity (Fig. 2d).

**Fig. 3:**
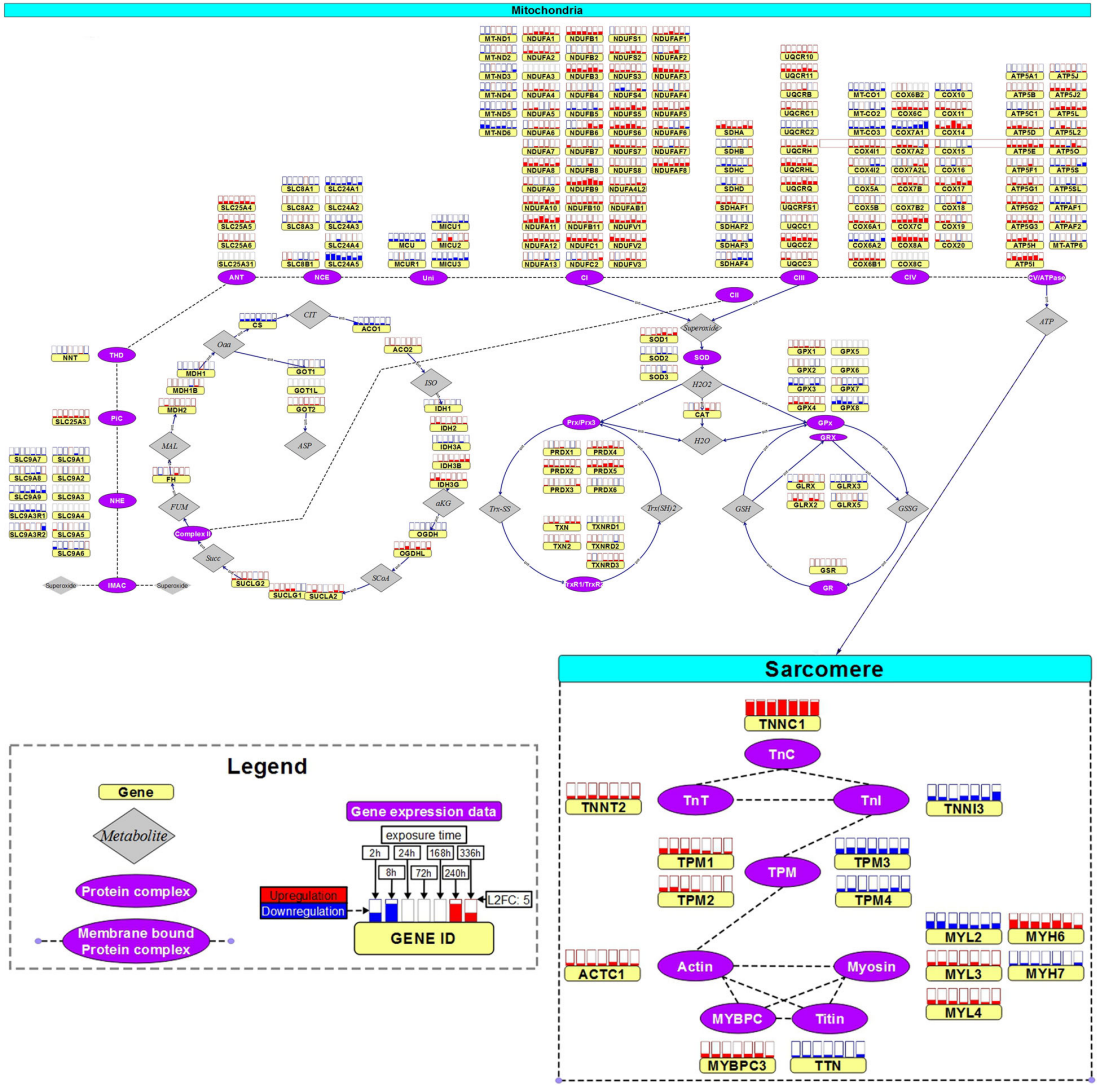
Dynamic transcriptome changes upon AC treatment in sarcomere and mitochondrion. Expression changes of mitochondrial response and sarcomere genes upon AC treatment at therapeutic dose measured with RNA-seq (expression changes with respect to toxic doses in Supplementary Fig. 8). Protein complexes are represented by purple, ovals with the genes encoding for subunits displayed next it in yellow rectangles. On top of each gene, the boxes display the expression change over time, where each box corresponds to a specific time point. The fill level of these boxes display the log2 fold change (completely filled boxes: log2 fold change ≥ 5), upregulations are depicted in red and downregulations in blue. Fold-changes were computed from the experimental replicates (*n* = 3) for each AC against the time-matched control samples. Color-codes refer to the average fold-change of the four ACs at the specific time point. Significance of temporal changes is indicated by transparency, where the lightest genes were not differentially expressed genes (DEGs) in any AC, medium transparent are DEGs with respect to only 1 AC treatment and not transparent are DEGs with respect to at least 2 AC treatments (figure adapted from Verheijen et al. ^87^).

On the transcriptome level, for each treatment and dose time-resolved RNA-seq analysis was performed and analyzed with longitudinal data analysis (Supplementary Methods) using a similar polynomial regression model approach as with the proteome data^35^. We found a total of 906 different dynamic response genes at both doses (DAU: 652, IDA: 388, EPI: 285, DOX: 204; Supplementary Data 3). The findings from the proteome and transcriptome analysis are consistent on the level of pathways (Supplementary Data 2 and 3). Pathway responses, in particular mitochondrial-related responses include those that were previously found with gene expression analysis in human pluripotent stem cell-derived cardiomyocyctes treated with substantially higher non-physiological DOX doses^36^. Functional enrichment of dynamic transcriptome responses cover sarcomere genes (*TNNT2*, *TNNC1*, *TPM1*, *TPM2*, *TPM3*, *TTN*, *MYL2*, *MYL3*, *MYH6*, *MYH7*), mitochondrial membrane proteins (*NDUFS6*, *COX6B2*, *PRKAR2B*, *CKMT2*) and electron transport chain components (e.g. *ATP5ME*, *COX6B1*, *NDUFA3*, *SLC25A5*). A summary of these transcriptome responses for all ACs is shown in Fig. 3.

### Integration of multi-omics data with network propagation amplifies functional content

We observe that AC responses at proteome, transcriptome and methylome levels enrich similar biological functions and pathways despite the fact that the molecular features driving this enrichment might be different. For example, all three sets of molecules enrich the *adrenergic signaling pathway* (Supplementary Fig. 9) although the molecular entities that participate in the enrichment are different in each omics data set. Thus, we conclude that different omics data deliver rather complementary information of the cell’s response network to drug treatment and that, in order to fully assess the integrated information, the molecular responses should be functionally interpreted on the level of networks. Since interacting proteins likely share function^37,38^, this prompted us to analyze the observed AC-induced dynamic responses in proteome and transcriptome in the context of protein interaction networks (Supplementary Fig. 10). We focused on these two layers because proteome and transcriptome express direct cellular metabolic and signaling responses to AC treatment that can be captured by PPI networks whereas the methylome rather displays cell identity and gene regulatory landscapes.

We have used 114,516 high-quality protein-protein interactions connecting 10,707 proteins from the ConsensusPathDB resource^39^ and populated the nodes in the PPI network with *p*-value scores that reflect the dynamic responses of the respective proteins/genes (see “Methods” section; Supplementary Fig. 11). Network propagation was computed by means of a random walk with restart approach adjusted from Hotnet2^21^, first for each single omics data set separately and then jointly for the integrated data sets (Supplementary Methods). For each AC and dosage network propagation resulted in the computation of subnetworks that agglomerate the major dynamic drug responses over time (Supplementary Figs. 12 and 13). We observe that the subnetworks computed from the integrated data contained the largest number of proteins followed by those computed from the proteome data. Subnetworks computed from the transcriptome were typically smaller (Supplementary Data 4; Supplementary Fig. 14). Furthermore, subnetworks derived from the integrated approach, combining transcriptome and proteome data, increased the functional content compared to the subnetworks derived from single omics layers (Supplementary Fig. 15).

**Fig. 4:**
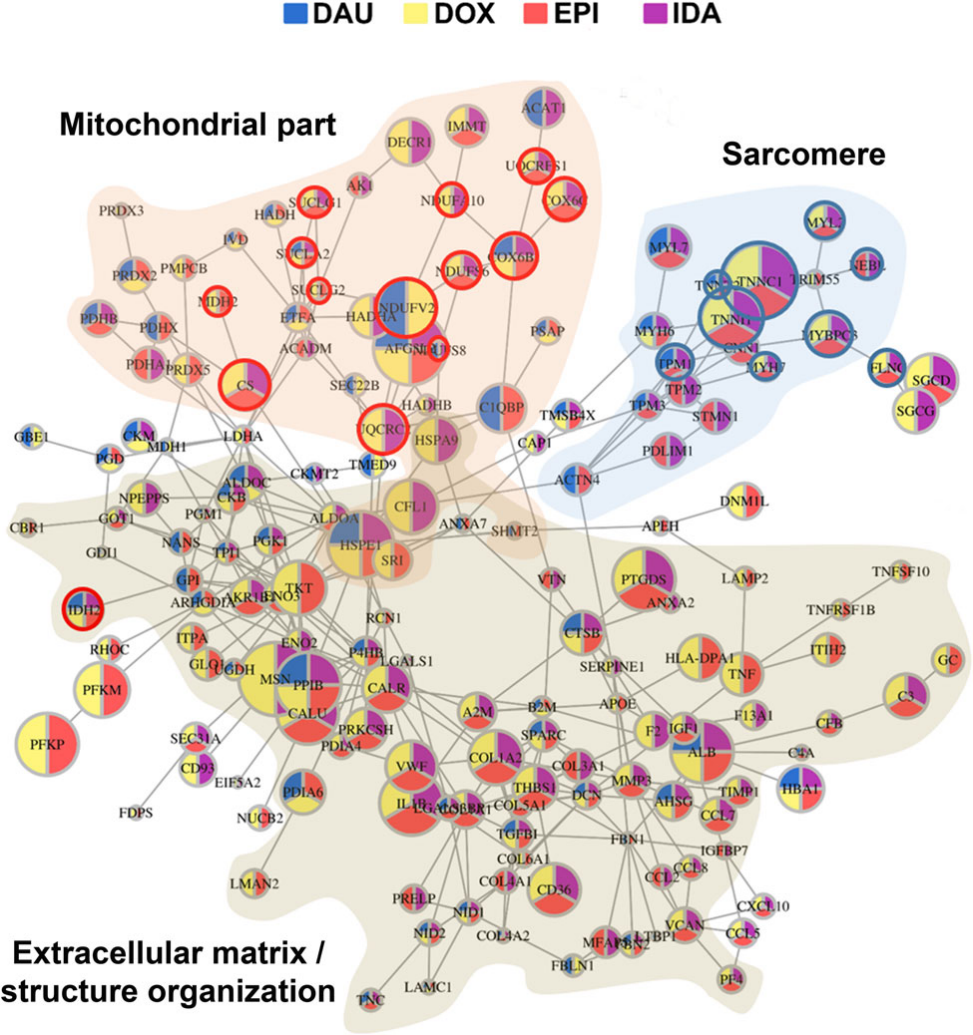
ACT response network. ACT response network computed from integrated proteome and transcriptome data (network derived from toxic doses is described in Supplementary Fig. 16). Nodes were initialized based on the significance of the dynamic changes of respective AC treatment compared to DMSO control longitudinal data and for each AC a drug response network was computed (Supplementary Fig. 12). Nodes and their interactions that appeared in at least two of the individual AC networks were integrated. Node colors reflect the occurrence of the node in the individual AC networks. The three major enriched cellular compartments are shown in transparent brown (extracellular matrix), transparent orange (mitochondrial part) and transparent blue (sarcomere). Red circles: Proteins that are contained in the mitochondrial model (Fig. 6a). Blue circles: Proteins genetically associated with dilated cardiomyopathy according to Talay et al.^40^.

Because we were mainly interested in the common AC responses at clinical conditions we continued with the four AC-subnetworks computed from the integrated data at therapeutic doses and combined these to an ACT response network that consists of 175 proteins (Fig. 4; Supplementary Methods). The ACT response network represents three major cellular compartments (cf. “Discussion” section): extracellular space, mitochondrial part and sarcomere. The mitochondrial part reflects the role of energy metabolism and ATP production (glycolysis, oxidative phosphorylation, TCA cycle, electron transport chain, HIF-1 alpha signaling), the extracellular space the role of extracellular matrix remodeling in heart diseases (ECM receptor interaction, focal adhesion, and TNF receptor pathways), and the sarcomere pinpoints cardiac disease pathways associated with contractility ability (dilated/hypertrophic cardiomyopathy, adrenergic signaling in cardiomyocytes, viral myocarditis).

### In vitro-derived ACT response proteins are expressed in patient biopsies and correlate with LVEF

We observed that proteins of the in vitro-derived ACT response network have high clinical relevance, for example *TNNC1*, *TNNT2*, *TPM1*, *MYH7, MYL2, FLNC, MYBPC3,* and *NEBL* genetic variants have been strongly associated with dilated cardiomyopathies as shown in a recent review^40^ (Supplementary Fig. 17).

**Fig. 5:**
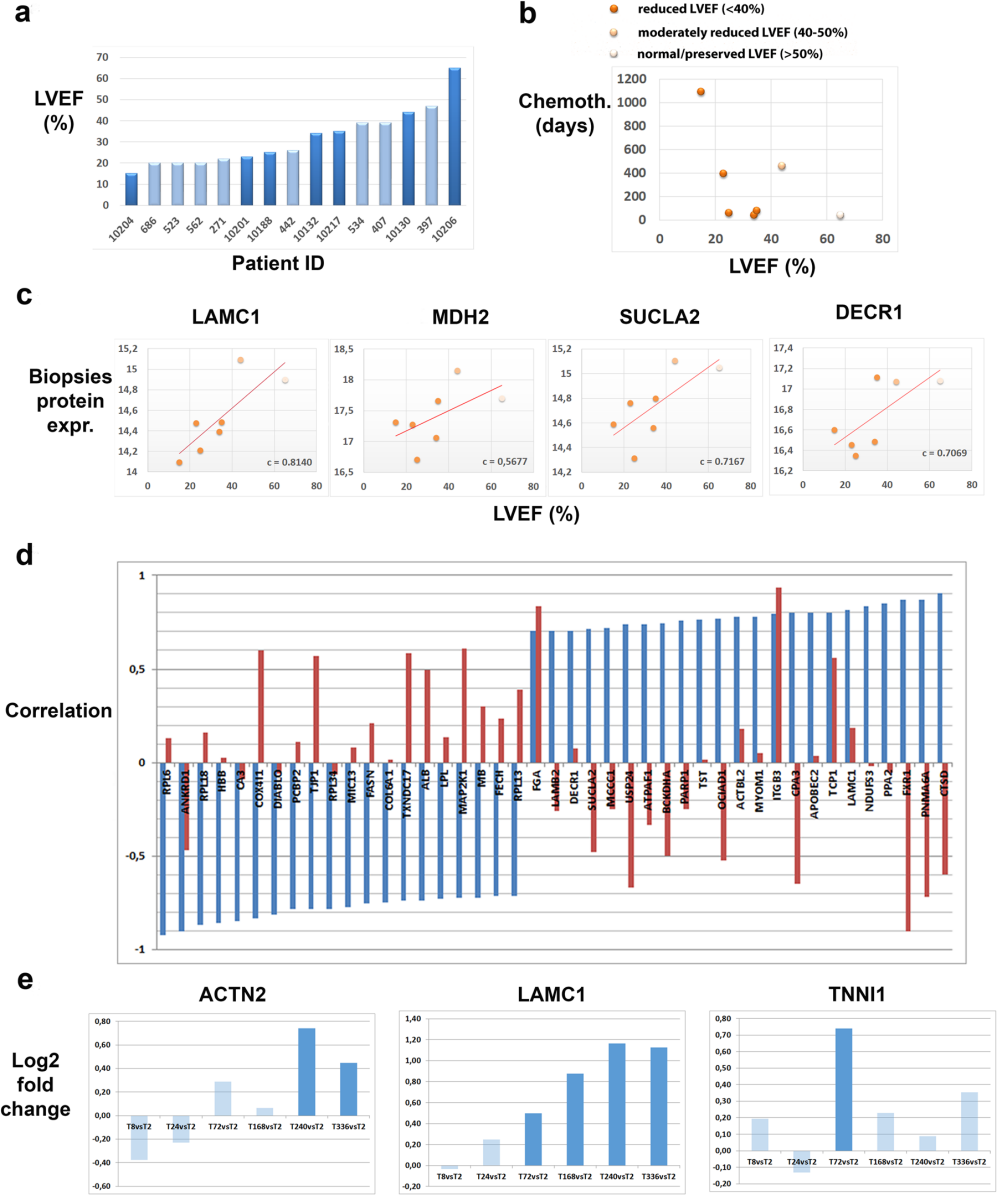
Clinical relevance of in vitro-derived proteins. **a** Patient characteristics. Histogram of LVEFs for the patients under study (*n* = 15). Chronic cardiotoxic patients (dark blue) and control cardiomyopathy patients (light blue) are sorted after LVEF. **b** LVEF (*X*-axis) of chronic cardiotoxic patients decreases with duration of chemotherapy in days (*Y*-axis); color of the nodes correspond to LVEF classification of the respective patient. **c** Examples of in vitro-derived ACT response proteins whose expression in biopsies of cardiotoxic patients correlate with LVEF. *X*-axes shows LVEF, *Y*-axis shows normalized protein expression. Color of the nodes correspond to LVEF status similar to **b**. **d** Discordant correlation values of biopsies protein expression with the LVEFs of the two patient groups. Blue bars: correlation with respect to cardiotoxic patients, red bars: control group. *X*-axis: genes, *Y*-axis: correlation values (range [−1,1]). **e** Pairwise comparison of protein expression fold-changes of measured time points (8, 24, 72, 168, 240, and 336 h) against the earliest time point (2 h) in 3D cardiac microtissues using Student’s *t*-test on *n* = 3 experimental replicates. *Y*-axis: bars indicate log2-fold change. Colors of the bars indicate significance of fold-change (dark blue: *Q* < 0.05; light blue: *Q* ≥ 0.05).

In order to evaluate the translational impact of the 175 ACT response network proteins we have performed LC–MS protein expression analysis of biopsies of 15 patients with cardiomyopathies (Supplementary Data 5). In a subset of these patients (*n* = 7) the cardiomyopathies were a consequence of prior anti-cancer therapy. The remaining patients (*n* = 8) had developed cardiomyopathies because of other reasons not related to drug toxicity. The major clinical indication of cardiotoxicity is decrease of the LVEF. We observed that on average the LVEF is not different between the two patient groups (Fig. 5a). However, for the patients suffering from cardiotoxicity the LVEF decreases with the length of therapy and thus reflects that the risk for cardiotoxicity can be caused by cumulative drug exposure^41^ (Fig. 5b).

121 (70%) proteins from the in vitro-derived ACT response network (Fig. 4) were identified in patient biopsies. In order to assess the clinical relevance of the in vivo-identified proteins we correlated their expression in the biopsies with the respective LVEFs of the chronic cardiotoxic patients. Highly correlating proteins include sarcomere proteins *ACTN2* (*c* = 0.61), *MYOM1* (*c* = 0.78), *TNNI1* (*c* = −0.59), members of the electron transport chain *COX4I1* (*c* = −0.84), *NDUFV1* (*c* = 0.61) and mitochondrial-related proteins *SUCLA2* (*c* = 0.72), *DECR1* (*c* = 0.71), *MDH2* (c = 0.57) (Fig. 5c). Among the highest correlation of protein expression with LVEF was observed for *LAMC1* (*c* = 0.81), a protein not predominantly known in the context of cardiotoxicity and thus representing a promising candidate for diagnosis. Laminin Subunit Gamma 1 (*LAMC1*) is a member of the *ECM-receptor interactions* and *Focal adhesion pathways* that have been associated functionally with cardiomyopathy previously (cf. “Discussion” section). Interestingly, the high correlation of the protein expression of these genes with the LVEFs of the chronic cardiotoxic cancer patients is in most cases accompanied by a low or even opposite correlation with the LVEFs of the control group of cardiomyopathy patients (Fig. 5d). This suggests a typical molecular response pattern of ACT distinct from other pathological paths to cardiomyopathy that can be captured by the in vitro microtissue model and which is translatable to the human in vivo situation eventually for predicting cardiopathological risks upon anti-cancer therapy. Such patterns have been previously proposed for the pre-clinical practice, for example to deliver cardiac safety indices for drug development and clinical settings^42^.

Since the ACT response network was computed from the complete longitudinal data, we additionally used time-point specific analysis (Supplementary Methods) to inquire whether early or late protein expression changes in microtissues were mostly responsible for identifying the above listed molecular markers of chronic cardiotoxicity. We have thus compared the expression of the proteins across all time points in the microtissues with the earliest time point (2 h) using Student’s *t*-test and observed that rather the intermediate and late time points contribute to the changes in protein expression of those proteins in vitro which translate to protein markers of AC-induced cardiotoxicity in cardiomyopathy patients. For example, upon DOX treatment at therapeutic dose we observe, among others, significant late responses (*Q* < 0.05) of *ACTN2* (240 and 336 h) and *LAMC1* (168, 240, and 336 h) in contrast to *TNNI1* which has an intermediate peak response at 72 h (Fig. 5e).

### Computational modeling of the physiological effect of ACT response network proteins on the mitochondrion

**Fig. 6:**
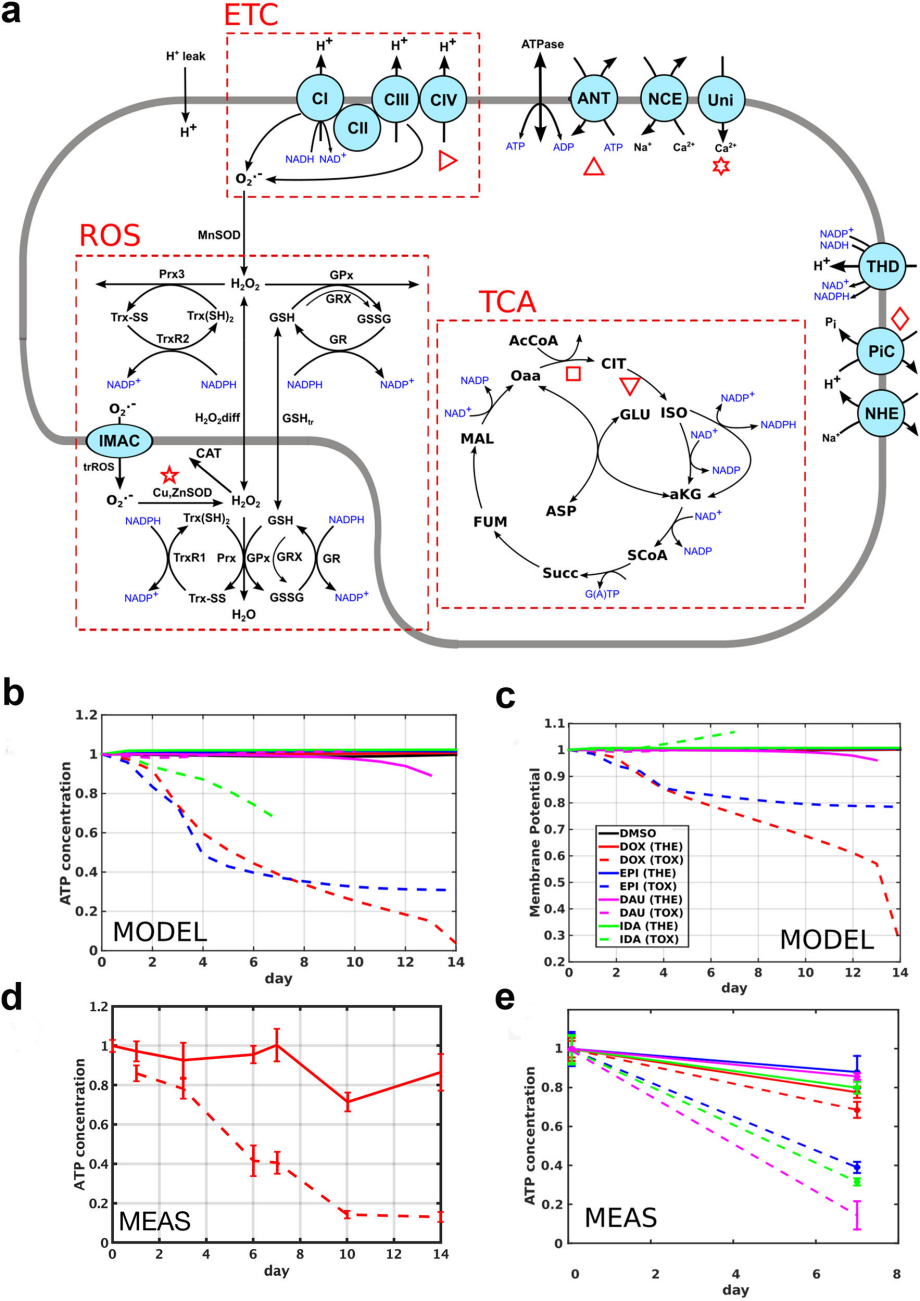
Computational modeling of in vitro-derived mitochondrial protein expression. **a** Mitochondrial model schematic, with subsystems indicated with the dashed boxes: electron transport chain (ETC), reactive oxygen species scavenging (ROS), and the tricarboxylic acid cycle (TCA). **b**, **c** Model predictions for the steady-state mitochondrial ATP concentration and membrane potential, as functions of exposure time, normalized to their initial value, for each drug under therapeutic (solid curves) and toxic doses (dashed curves). **d**, **e** Functional measurements of ATP concentration performed on spheroid microtissues at discrete time points, following either therapeutic (solid lines) or toxic (dashed lines) drug exposure as implemented using a PBPK model. MEAS measured, MODEL predicted by model.

To provide physiological context for protein expression, we evaluated the effect of measured changes in expression of predicted mitochondrial function in a biophysical model. In vitro-derived dynamic protein fold changes corresponding to mitochondrial proteins were mapped onto a biophysical model encoding 181 proteins responsible for ROS scavenging, the electron transport chain and the TCA cycle^43,44^ (Fig. 6a). The effect of the protein fold changes on ATP homeostasis and membrane potential over time for the four ACs at toxic and therapeutic doses and DMSO controls were predicted (Fig. 6b, c). ATP loss increases with dose which is consistent with repeat experimental measurements (see “Methods” section; Fig. 6d, e).

We contrasted the ACT response network proteins (Fig. 4) with components in the mitochondrial model that have an impact on ATP concentration as measured with sensitivity analysis. We found that relevant model components are in fact covered by the drug response network. For example, seven model components have been identified to significantly impact ATP concentration after DOX treatment by computational sensitivity analysis (Supplementary Fig. 18). Five out of these seven model components are covered by proteins of the drug response network, in particular TCA cycle proteins (*CS*, *MDH2*, *SUCLA2*, *SUCLG1*, *SU*CLG2) and electron transport chain proteins (*NDUFV2*, *UQCRC1*, *UQCRC2*, *UQCRFS1*). For the other ACs, we found six out of seven significant model components being covered by network module proteins for EPI, seven out of nine for IDA and three out of four for DAU. These covered components relate mostly to the TCA cycle and the electron transport chain (Supplementary Fig. 19). This demonstrates that the integrated network propagation approach is able to identify physiologically relevant proteins that have a significant impact on ATP production and thus, mitochondrial function in cardiomyocytes.

## Discussion

Previous studies of anthracycline toxicity have characterized organ-scale phenotypes^45,46^ or focused on specific individual mechanisms. Broad ex vivo omics studies have identified potential candidate proteins and pathways but have not confirmed these in patient studies^47–50^. Conversely, patient biopsy studies in isolation of targeted ex vivo experiments are at risk of being confounded by multiple drug exposures and co-morbidities^51,52^. Our study is the first multi-omics network analysis to show that a spectrum of anthracyclines do not target a single molecular pathway but act across multiple critical cellular systems, including mitochondrial, extracellular matrix and sarcomere proteins in a human ex vivo system, and critically we confirm the importance of these pathways in patient biopsy studies.

The dominant pathway changes may be attributable to direct action of anthracyclines or may be a secondary change due to anthracycline action on one or more of the other pathways. Specifically, anthracyclines are known to act on the mitochondria increasing ROS production, which can modulate downstream protein expression. Increased ROS production alters the beta-adrenergic signaling pathways, sarcomere proteins, and extracellular matrix synthesis^53^. ROS is known to bind to *DES* (desmin), MHC genes, *TTN* (titin), *TNNC1*, among others^54^ which has a high level of overlap with the inferred proteome and transcriptome changes. Changes in extracellular matrix/structure organization protein regulation are consistent with observations in patients^55^ and mice^56^ that find increased fibrosis following anthracycline exposure. The fibrosis may be reparative in response to apoptosis or interstitial in response to changes in loading. Changes in extracellular matrix/structure organization proteins may be due to direct effects of anthracyclines or secondary due to increased ROS^57^ or changes in mechanical loading^58^. Of the proteins associated with LVEF in biopsies we identified the extracellular matrix protein *LAMC1*, consistent with either a dominant or integrating effect of the extracellular matrix in determining LVEF in cardiotoxic patients.

While our primary focus was on extracting commonalities of AC responses, we see also clear differences with different ACs. A particular issue is whether the level of cardiotoxicity of the individual ACs can be extrapolated from the proteome data. We have several indications (based on the number of dynamically changed proteins, the sizes of the computed integrated networks, the enrichment of cardiovascular disease pathways) that the most plausible order of toxicity is DOX > EPI > IDA > DAU. This is in line with in vivo-derived phenotypic observations: for example, Platel et al. ^59^ showed that, in rat at maximum tolerated doses, IDA showed significantly lower cardiotoxicity than DOX. Furthermore, the computer simulations predicted differences in protein changes between different ACs. Despite similar predicted changes in membrane potential and ATP for DOX, EPI, and IDA, the cause was distinct between compounds. DOX increases ETC and TCA fluxes; conversely IDA and EPI cause a decrease in TCA and ETC fluxes (Supplementary Fig. 20a). The sensitivity of ATP concentration to small perturbations in protein densities was calculated for the therapeutic and toxic dosing regimens (Supplementary Fig. 20b). This identified that all proteins had a similar effect on ATP concentration for IDA, EPI, and DAU, however, toxic DOX dosing caused a shift in the operational space of the mitochondria with an increased dependence of ATP concentration on TCA flux and ROS scavenging. Simulations were then performed where the impact of each drug on each protein was sequentially removed from the model to provide a ranked list of the role of each protein in determining ATP concentration. This rank was compared with the fold change to show that large changes in protein abundance do not always coincide with an important functional role (Supplementary Fig. 20c).

This study has generated several novelties. Firstly, using integrated network modeling we were able to identify potential biomarkers that translate from in vitro microtissues to patient biopsies and that are indicative of clinical parameters (Fig. 5b, c). This is important for drug development and personalized medicine since these biomarkers can be used to monitor cardiotoxicity of drug effects before entering clinical phase on the one hand and on the other hand soften or preventing cardiotoxicities of anti-cancer drug therapies.

Secondly, this study is the first in-depth study of AC effects on whole-genome methylation in human cardiomyocytes. We have shown that in our human iPSC-derived microtissues, gene body methylation is inversely correlated with gene expression, a result that had been found previously in human and murine adult cardiomyocytes^26,27^. Furthermore, it has been observed in murine cardiomyocyctes that this gene body methylation of key sarcomere genes, such as troponin I isoforms (*Tnni1* and *Tnni3*) is highly dynamic^26^: While *Tnni1* gene body was de novo methylated in adult murine cardiomyocytes, *Tnni3* was demethylated. In our microtissues we observed that *TNNI1* is methylated in cardiac microtissues throughout the gene body while *TNNI3* has a significant reduction in methylation at exons 4 and 5 (Supplementary Fig. 21).

Furthermore, we found binding sites of key regulators of cardiac dysfunction such as *YY1* and *SRF* enriched in hyper-methylated promoters after AC treatment suggesting interference of regular gene regulation (Fig. 1c). In addition, we found the target sets of these regulators enriched among the dynamically altered AC response proteins (Fig. 1d). It was shown previously that *YY1* functions as an anti-hypertrophic factor and up-regulation of *YY1* in human heart failure could be a protective mechanism against pathological hypertrophy^60^. Furthermore, *Yy1* in rodents acts as a suppressor of DCM and cardiac fibrosis through regulation of *Bmp7* and *Ctgf*^61^, as well as a suppressor of DCM caused by *Ttn* insufficiency^62^. In addition, *SRF*-dependent gene expression was modulated during heart failure in human patients as well as rodent models^63^.

A striking observation of the methylation analysis is that more DMRs were identified with therapeutic than with toxic doses for EPI, IDA, and DAU (Supplementary Figs. 4 and 5). This seems counterintuitive, however, might be caused by a demethylation mechanism in a fraction of the cells that is induced by increased oxidation of 5mC sites. ACs are known to induce oxidative stress, and ROS may oxidize the 5mC sites leading to increased levels of hydroxymethylated DNAs (5hmCs) at toxic doses what subsequently could lead to increased demethylation via the TET pathway^64^.

Thirdly, dynamic network modeling identified proteins that are typically not easy to identify with MS-approaches such as transcription factors. Here, we showed evidence for a role of TRIM proteins in AC cardiotoxicity, in particular *TRIM55* and *TRIM28* (*Tripartite motif-containing proteins 55 and 28*) that belong to the superfamily of TRIM proteins that function as regulators for a variety of human diseases^65^. *TRIM55* is also known as Muscle-specific Ring Finger protein 2 and has been shown to localize to the sarcomere and to regulate muscle protein turnover^66^. *TRIM55* has several connections to sarcomere genes (Fig. 4) and thus has been predicted as ACT response protein through network propagation despite the fact that the MS profiles have many missing values so that the protein has not been fully identified by LC–MS analysis. *TRIM28* profiles after DOX, DAU (Fig. 2b), and IDA treatment were significantly altered compared to the DMSO control experiments. Furthermore, the TRIM28 target set was enriched by the entire set of 641 dynamic response proteins (enrichment *Q*-value *Q* = 0.054).

In conclusion, our study proofs iPSC-derived 3D cardiac microtissues as a suitable in vitro cell model for screening dynamic drug responses at multiple molecular layers. We have identified a set of 641 proteins and 904 transcripts that are dynamically changed over time when compared to DMSO control experiments. Although the overlap across these data sets is fairly low, the biological pathways and functions that are enriched by the different molecular layers are similar. This prompted us to integrate molecular data in a PPI network with *p*-value scoring and to perform network propagation analysis in order to compute for each drug a subnetwork accumulating the major dynamic responses from the integrated data sets. This approach is rather generic and can be applied to various other data (such as mutations, GWAS results, clinical data) as long as the results can be quantified by *p*-values. It thus might serve as a template for extracting common information from complex multi-omics data sets, likely to be massively generated in future precision cardio-oncology^67^. We combined the four individual drug subnetworks at therapeutic doses to an ACT response network consisting of 175 proteins and showed that 70% of these proteins were in fact expressed in cardiac biopsies of patients and that potential biomarkers inferred from the network analysis correlate with pathological parameters and mechanisms.

## Methods

### Experimental dosing scheme

A previously established workflow for model-based assay design was used to reproduce in vivo patterns of drug-induced toxicity in spheroids. For each of the four anthracyclines physiologically based pharmacokinetic (PBPK) models were developed. The models were built with the open source PBPK modeling software PK-Sim and validated according to best practice guidelines for PBPK model qualification^18,68^.

Each PBPK model was used to simulate in vivo drug exposure in the interstitial space of the heart following administration of either a therapeutic or toxic drug dose over 2 weeks of a once daily administration schedule. Here, the therapeutic dose was based on a standard clinical dosing regimen according to the specific drug label. Toxic doses were estimated based on in vitro viability experiments in 3D cardiac microtissues (IC20). A toxic dose for each drug was then calculated from the IC20 values by reverse dosimetry. To this end it was assumed that the unbound drug exposure in the interstitial heart compartment of the PBPK model was equal to the in vitro drug. Finally, the continuous exposure profiles of the PBPK models where translated into an experimental setting with three daily media changes mimicking the estimated in vivo PK exposure profile (Supplementary Methods; Supplementary Fig. 2).

### Cardiac 3D microtissues treatment

Commercially available human iPSC-derived cardiomyocytes were obtained from Cellular Dynamics International Inc. (CDI, Madison, WI, USA). Shortly, these iCell cardiomyocytes were derived from an engineered hiPSC clonal line reprogramming human fibroblasts from a female Caucasian donor as described in Ma et al. ^69^. The iPSC-derived cardiomyocytes were aggregated into 3D cardiac spheroids as originally described23. Human 3D cardiac microtissues (InSphero, SWL) were used, containing ~4000 iPSC-derived human cardiomyocytes (female Caucasian donor) and 1000 cardiac fibroblasts (male Caucasian donor) per microtissue. The microtissues were cultured in 50 μl per well 3D Insight™ Human Cardiac Microtissue Maintenance Medium (InSphero, Cat #CS-07-010-01).

To accomplish the PBPK-based repetitive dosing profile, the medium of the microtissues was changed three times daily on working days at intervals of 2, 6, and 16 h with the PBPK-determined concentrations (Supplementary Table 1). These were administered using stock solutions of the compound dissolved in DMSO. At the time of redosing 50 μl of media was aspirated from each well using an electronic 96-channel pipette and replaced with 50 μl fresh media with the adapted anthracycline concentration. Final DMSO percentage did not exceed 0.1%. As control, microtissues exposed to similar end concentrations of DMSO were used. Seven time points (2, 8, 24, 72, 168, 240, and 336 h) were sampled in triplicates during the 2-week treatment period. For each sample, 36 microtissues were incubated separately, subsequently pooled, snap-frozen in liquid nitrogen and stored at −80 °C. Genetic material was extracted manually with the Qiagen’s AllPrep DNA/RNA/miRNA Universal Kit (Cat#80224) which was used according to the manufacturer’s protocol.

### Patient biospies

All patients that underwent endomyocardial biopsies (EMB), first had a physical examination, blood sampling, 12-lead electrocardiogram, 24-h Holter monitoring on indication, and a complete echocardiographic and Doppler evaluation. Significant coronary artery disease as a cause of the decreased ejection fraction was excluded by a coronary angiography (CAG) or a CT-angiography at baseline. EMB were performed as part of routine diagnostic work-up in non-ischemic, non-valvular cardiomyopathy, upon consent of the patient, as part of the Maastricht Cardiomyopathy Registry with inclusion and exclusion criteria as described previously^70^. The main indication for EMB was a LVEF < 45% after 6 months of optimal medical treatment, and the absence of other.

In short, both DCM and hypokinetic non-dilated cardiomyopathy (HNDC; also called isolated LV dysfunction) according to the latest ESC proposal were included (DCM defined as LVEF < 50% with an indexed left ventricular end diastolic diameter (LVEDDi) >33 mm/m^2^ (men) or >32 mm/m^2^ (women) measured by echocardiography; and HNDC defined as LVEF < 50% with an LVEDDi ≤ 33 mm/m^2^ (men) or ≤32 mm/m^2^ (women) measured by echocardiography in the absence of a (i) myocardial infarction and/or significant coronary artery disease; (ii) primary valvular disease; (iii) hypertensive or congenital heart disease; (iv) acute myocarditis; (v) arrhythmogenic right ventricular dysplasia; and (vi) hypertrophic, restrictive or peripartum cardiomyopathy. For the present study, we included cases with a previous history of cardiotoxic-anthracycline chemotherapy, and control DCM/HNDC without. The study was performed according to the declaration of Helsinki and was approved by the Medical Ethics Committee of Maastricht University Medical Centre. All patients gave written informed consent.

### Proteomics sample preparation

Cardiac spheroids and cardiomyocytes were resuspended in 100 μl lysis buffer containing 8 M urea, 1 mM dithiothreitol, 0.1 M ammonium bicarbonate, pH 7.8. After four freeze–thaw cycles, the samples were centrifuged at 16,000×*g* for 15 min at 4 °C and protein concentrations were assessed with the Qubit™ Protein Assay Kit (Invitrogen, Molecular Probes). Protein isolates were then submitted to in-solution digestion^71^ or filter aided sample preparation (FASP)^72^. Protein digestions were stopped by adding formic acid to a final concentration of 1%. The peptides were cleaned up using Sep-Pak tC18 cartridges (Waters) according to the manufacturer’s instructions, and eluted with 60% ACN and 0.1% formic acid (Sigma-Aldrich, USA). Patient biopsies were prepared as described in Guo et al. ^73^.

### Proteomics mass spectrometry measurements

Samples were submitted to an Orbitrap Fusion mass spectrometer (Thermo Fisher Scientific) coupled to a NanoLC-2D HPLC system (Eksigent, Dublin, CA) or EASY-nLC 1000 system (Thermo Fisher Scientific, Germany). Samples were loaded onto a self-made column (75 μm × 150 mm) packed with reverse-phase C18 material (ReproSil-Pur 120 C18-AQ, 1.9 μm, Dr. Maisch HPLC GmbH) when coupled with the EASY-nLC 1000 system and onto an Easy-Spray Column (75 μm × 500 mm) packed with reverse-phase C18 material (Silica 100 Å, 2 μm) when coupled with the NanoLC-2D HPLC system. Peptides were separated with a linear gradient of acetonitrile/water, containing 0.1% formic acid, at a flow rate of 300 nl/min. A gradient from 5% to 30% acetonitrile in 60 min was used. The mass spectrometer was set to acquire full-scan MS spectra (300–1500*m*/*z*) at 120,000 resolution at 200*m*/*z*; precursor automated gain control (AGC) target was set to 400,000. Charge-state screening was enabled, and precursors with +2 to +7 charge states and intensities >5000 were selected for tandem mass spectrometry (MS/MS). Ions were isolated by use of the quadrupole mass filter with a 1.6*m*/*z* isolation window. Wide quadrupole isolation was used, and injection time was set to 50 ms. The AGC values for MS/MS analysis were set to 5000 and the maximum injection time was 300 ms. HCD fragmentations were performed at a normalized collision energy (NCE) of 30%. MS/MS were detected in the ion trap in centroid mode. Precursor masses previously selected for MS/MS measurement were excluded from further selection for 25 s, and the exclusion window was set at 10 ppm.

### Proteomics data processing and normalization

Raw MS data were processed using Genedata Expressionist^®^ software v.11.0, consisting of two modules: Refiner MS (data pre-processing) and Analyst (data post-processing and statistical analysis). In short, after noise reduction and normalization, LC–MS peaks were detected and their properties calculated (*m*/*z* and RT boundaries, *m*/*z* and RT center values, intensity). Individual peaks where grouped into clusters and MS/MS data associated to these clusters were annotated with MS/MS Ions Search (Mascot 2.6) using peptide tolerance: 10.0 ppm, MS/MS tolerance: 0.50 Da, max missed cleavages: 2 and database: Uniprot Swiss-Prot 29062016, Taxonomy *Homo sapiens* (human). Results are validated by applying a threshold of 5% normalized false discovery rate (FDR). Protein interference was done based on peptide and protein annotations. Redundant proteins were ignored according to the Occam’s razor principle, and at least two peptides were required for a positive protein identification (shared peptides were ignored). Protein intensities were computed using the Hi3 method. A maximum of the top 3 peptides per protein (based on the average intensity across samples) was used in the calculation. If a peptide was identified in multiple charges (2+, 3+, 4+) and modification states (carbamidomethyl (C), deamidated (NQ) or oxidation (M)), values were consolidated into a single peptide intensity. The volume of a peak is computed as the area under the intensity curve inside the peak region. The area under the intensity curve is subdivided into trapezoids at the data points according to the trapezoidal rule. After the data pre-processing, the intensities were log2 transformed. Normalization was performed as follows: (i) the transformed data of the DMSO (control) samples were shifted to the median of the medians determined by a reference group consisting of the proteins found in all these control samples, (ii) for every treatment/dose combination and for each time-point the common protein set between the controls and the treatment samples was determined, (iii) the median of the medians of the (in general 3) normalized control samples was determined using this common protein set between the controls and the treatment samples, and (iv) the data from the samples of the treatments were shifted to these medians. Two-sided Student’s *t*-tests were then used for the determination of differentially expressed proteins (DEPs) comparing the proteins of each time-point and dose against the corresponding time-matched control. These normalized data sets were also used for the two-step regression model in order to identify temporal protein expression changes.

### RNA-seq data generation

Total RNA was isolated for each exposed microtissues using Qiagen AllPrep DNA/RNA/miRNA Universal Kit (Cat #80224). Sample were depleted of ribosomal RNA using the Illumina RiboZero Gold kit (Cat #MRZG12324) and libraries were prepared for sequencing using Lexogen SENSE total RNA library preparation kit (Cat #009.96). The samples were sequenced on the HiSeq2500 (100 bp paired-end).

### RNA-seq data analysis

Raw RNA-seq data were processed using Genedata Profiler^®^ software v.11.0. The first 12bases of the 5′end of all reads and adapter sequences were removed using Trimmomatic version 0.32^74^. Data quality was checked using FastQC before and after trimming. Sequencing reads were mapped to the human genome version hg38 with the splice junction mapper STAR (version 2.5.3a)^75^ using as annotation the reference genome gencode version 26 (October 2016 freeze, GRCh38)—Ensembl 88. Quantification of genes or transcripts, respectively, was performed with an algorithm based on Cufflinks^76^. Features used for quantification were protein coding and non-protein coding sequences (e.g. pseudo-genes missing a CDS of the transcripts). Differential expression analysis of the RNA-seq experiments was performed with DESeq2 (version 1.14.1)^77^. The quality of the samples was assessed according to the amount of (mapped) reads, Cook’s distance, hierarchical clustering, principal component analysis, and sample dispersion. Outliers were excluded from further analyses (Supplementary Methods). For the comparison of each time-point of a treatment with the corresponding time-matched DMSO control input matrices for DESeq2 consisted of the samples from DMSO and the samples from a treatment with either the therapeutic or the toxic dose. Comparisons were finally done applying the ‘contrast’ argument in DESeq2.

### Dynamic longitudinal analysis of proteome and transcriptome data

We have used MaSigPro (version 1.46.0) to calculate for each gene or protein, the fit of the quadratic regression model (degree = 2):

1 $$y_{ijr} = \left( {\beta _{0,C} + \beta _{0,{\mathrm{TvsC}}}} \right) + \left( {\beta _{1,C} + \beta _{1,{\mathrm{{TvsC}}}}} \right)t_{ijr} + \left( {\beta _{2,C} + \beta _{2,{\mathrm{{TvsC}}}}} \right)t_{ijr}^2 + \varepsilon _{ijr}$$

Here *i* = 1,2 describes the treatment/control conditions, *j* = 1,..,7 the different time points and *r* = 1,2,3 the replicate experiments. *β*_0,TvsC_, *β*_1,TvsC_, *β*_2,TvsC_ describe the regression coefficients for the constant, linear, and quadratic terms and the corresponding *p*-values, *p*_0,TvsC_, *p*_1,TvsC_, *p*_2,TvsC_, describe the deviation from the control experiment^33,35^. Thresholds for the analyses were set to a Benjamini–Hochberg (BH) *q*-value of ≤0.05, an *R*-square of the regression model of ≥0.7, and a minimal observation number of 10. For the two-step regression model searching for significant different time-dependent gene expression profiles across the analytical groups, the input matrices for DESeq2 consisted of all samples from the controls and the treatment/condition groups (therapeutic and toxic) and the DESeq2-derived rlog-transformed data were then used for the analysis of the time-dependent gene expression profiles. For the analysis of the time-dependent protein expression profiles the previously described normalized data sets were used as input. For each treatment and dose the analysis was carried out separately, the resulting dynamic response genes/proteins are summarized in Supplementary Data 2 and 3).

### MeDIP-seq data generation

For preparation of microtissue MeDIP-Seq libraries, a previously published low input MeDIP protocol^78^ was modified. DNA was fragmented to 100–200 bp using the Covaris S2 system. Because of low DNA yield for DOX and IDA samples, the triplicate samples were pooled before fragmentation. End repair and A tailing was performed using the NEBNext^®^ Ultra™ library prep kit for Illumina^®^ (NEB), adapters were ligated with NEBNext^®^ Ultra™ Ligation Module (NEB) and samples were purified using Agencourt^®^ AMPure^®^ XP beads (Beckman Coulter). Methylated fragments were captured using the MagMeDIP kit (Diagenode). In short, denatured DNA was mixed with anti-5-meC-antibody and captured using magnetic beads. Capture efficiency was determined by qPCR against spiked-in Lambda-DNA fragments in precapture and postcapture library samples. Libraries were amplified in a final PCR step using barcoded TruSeq primers. Quality was assessed on Agilent Bioanalyzer 2100 and library concentration was determined by Qubit™ and qPCR.

### MeDIP-seq data analysis

In order to gain exhaustive genome-wide coverage the triplicate samples that have been sequenced individually were merged before alignment. MeDIP sequencing reads were aligned to the GRCh38 reference genome using bwa Version 0.7.15-r1140^79^, and analyzed in 250 bp windows using the R/bioconductor package QSEA^25^ with standard parameters. Within QSEA, the MeDIP enrichment was calibrated with 450k methylation array measurements of primary hepatocytes (GSM999339) and cardiac myocytes (HCM, GSM999381) from ENCODE^80^, for the hepatic and cardiac micro-tissues, respectively. To this end, beta values of the calibration samples were computed with the R/Bioconductor package Minfi^81^, genomic locations of the array probes were mapped from GRCh37 to GRCh38 using the UCSC liftOver command line tool^82^, and probes within 250 base windows were averaged. DMRs obtained from QSEA were annotated with gene, exon, and promoter (transcription start site ±2 kilobases) information from RefSeq, ENCODE TFBS and model-based CpG islands, all obtained via the UCSC table browser. Since ENCODE TFBS were not available for GRCh38, genomic locations were mapped from GRCh37 using the liftOver tool.

### Network propagation

*Interaction network generation*: We agglomerated a large PPI from 19 different data resources^83^ which has been proven to be one of the most effective networks for identifying disease genes in a recent comparison^84^. We have performed quality assessment of the individual interactions using different supervised and unsupervised methods^85^ in order to generate a high-quality PPI network consisting of 10,707 proteins and 114,516 interactions.

*Node scoring*: In order to weight the proteins/genes according to their information content with respect to time-sensitive AC treatment responses we used scores that reflect the dynamic changes of the proteins after AC treatment: For each protein *i*, the fit of the quadratic regression model (above section) is described by the estimated regression parameters $\beta _{0,{\mathrm{{TvsC}}}}^{(i)},\,\beta _{1,{\mathrm{{TvsC}}}}^{(i)},\,\beta _{2,{\mathrm{{TvsC}}}}^{(i)}$ and the corresponding *p*-values, $p_{0,{\mathrm{{TvsC}}}}^{(i)},\,p_{1,{\mathrm{{TvsC}}}}^{(i)},\,p_{2,{\mathrm{{TvsC}}}}^{(i)}$ that describe the deviation from the control time course. The score for the protein is then computed as

2 $$S_i = - \mathop {\sum}\nolimits_j {\log _{10}} p_{j,{\mathrm{{TvsC}}}}^{(i)}$$

if the fit was significant and set to zero elsewhere. This score is computed for (i) proteome data only, (ii) transcriptome data only, and (iii) integrated data as the sum of proteome and transcriptome scores (Supplementary Fig. 11).

*Network propagation*: Network propagation was done with Hotnet2^21^ based on a version of the random walk with restart method (insulated heat diffusion). This method was developed initially for tracking the effect of mutations across a network. We adapted this approach to analyze the effects of proteome/gene expression data. For each longitudinal data series we initialized the nodes of the networks as shown above so that these initialized networks per AC, dose and data platform can be interpreted as the dynamically affected interactome. Network propagation then delivers a final ranking of all proteins (final scores) along with a subnetwork containing the major connected components of the drug response. Hotnet2 offers four different subnetworks depending on a threshold parameter *δ*, and in all cases we chose the subnetwork derived with the minimal threshold parameter.

### Computational modeling of mitochondrial functions

*Numerical simulations*: The mitochondrial model, represented schematically in Fig. 6a, is described with a system of coupled ordinary differential equations, following the principle of mass action, and is used as described in earlier studies^22,44^. The reactions in the system were grouped into three subsystems, relating to the electron transport chain (ETC), ROS scavenging, and the tricarboxylic acid cycle (TCA). The model solutions seek to emulate the functional behavior of the mitochondrion subject to drug exposure by modifying the kinetic parameters of the baseline model^22^ as deduced from the in vitro experiments conducted on 3D cardiac microtissues. The solution of the differential equations then yield the predicted flux through each reaction in the mitochondrial theoretical model, and the procedure was repeated for each drug and dosage in order to map the evolution of the steady state over up to 14 days, as per the in vitro experiments. The effect of a given drug and dose level on individual reaction rates, at each time point, was estimated experimentally by applying the drug to the microtissues, following a PBPK-based protocol. The drug exposure was then implemented in the simulations by scaling the appropriate reaction rates based on the predictions derived from the experimental time-series data, fitted to a polynomial model.

For each such reaction rate configuration, the set of coupled differential ordinary differential equations was solved using the *ode15s* numerical solver of Matlab until a steady state was reached (achieved by running the simulations over 50,000 time steps of 60 s). Average fluxes through the ROS, ETC, and TCA subsystems are plotted in Supplementary Fig. 20a for each drug and dose. Further readouts of the simulation output, the ATP concentration and membrane potential, are shown in Fig. 6b and c.

*Functional measurements*: The simulation results were compared with intracellular ATP concentrations measured in the microtissues using a CellTiter-Glo cell viability assay (Luxcel, Cork, Ireland). The amount of ATP detected in the assay increases with the number of viable cells in the culture, as described in other studies^86^. The assay is a luminescence test involving a detergent component that dissociates the cells and disrupts the membrane. The released ATP then catalyzes the luciferase reaction, making the luminescence intensity proportional to the amount of ATP, as determined by comparison with a standard curve.

*Sensitivity analyses*: sensitivity analyses were performed to assess the relative impact of the drug exposures to each reaction in the system (Supplementary Fig. 18). In the first analysis, the magnitude of each reaction in the scheme (Fig. 6a) was increased by 1% before allowing the system to settle to a steady state, for each anthracycline and each dose level in turn. The impact on the mitochondrial ATP concentration, expressed as a relative change per fractional increase in the reaction activities, was calculated at the 7-day time point (where all the simulations converged). The results are plotted in Supplementary Fig. 20b allowing a comparison of the therapeutic and toxic dose levels. To further estimate the effect of the drugs in this highly nonlinear system, we performed a complementary sensitivity analysis, by considering macroscopic changes to the reaction activities. By this approach, the sensitivity of the ATP concentration to each reaction in the model was calculated, in turn, by scaling the rates for all the reactions according to the toxic dose of each drug (at day 7), except for one reaction that was kept at its reference activity level, and comparing these simulation outputs to the case where all the reactions are altered. The results are plotted in Supplementary Fig. 20c, as functions of the protein fold change for the corresponding reaction.

### Reporting summary

Further information on research design is available in the Nature Research Reporting Summary linked to this article.

## Supplementary information

Supplementary Information

Description of Additional Supplementary Files

Supplementary Data 1

Supplementary Data 2

Supplementary Data 3

Supplementary Data 4

Supplementary Data 5

Reporting Summary

## Data Availability

Data have been submitted to the BioStudies repository (https://www.ebi.ac.uk/biostudies/) and is available under the following accession numbers: - Methylation data: S-HECA339, S-HECA340, S-HECA341, S-HECA343, S-HECA347, S-HECA352, S-HECA353, S-HECA363, S-HECA431, S-HECA432, S-HECA433, S-HECA434. - Proteomics data: S-HECA2, S-HECA3, S-HECA19, S-HECA20, S-HECA21, S-HECA22, S-HECA38, S-HECA39, S-HECA54. - RNA-seq data: S-HECA10, S-HECA11, S-HECA12, S-HECA148, S-HECA151.

## Code availability

The code for analyzing the methylome data is available through the R/Bioconductor package QSEA (http://bioconductor.org/packages/release/bioc/html/qsea.html), and the code for performing the longitudinal proteome and transcriptome data analysis through the R/Bioconductor package masigpro (https://www.bioconductor.org/packages/release/bioc/html/maSigPro.html). The code for processing and normalizing the proteomics and transcriptomics data is proprietary (Genedata Profiler^®^ software v.11.0). The code for performing the network propagation is available from github (https://github.com/raphael-group/hotnet2).
