# Supplementary material for: Network integration and modelling of dynamic drug responses at multi-omics levels
Source: Commun Biol. 2020 Oct 15;3:573. doi: 10.1038/s42003-020-01302-8 (PMC7567116; doi:10.1038/s42003-020-01302-8)
Supplement: Supplementary file 1 — Supplementary Information [file 42003_2020_1302_MOESM1_ESM.pdf]

Network integration and modelling of dynamic drug responses at multi-omics levels

Nathalie Selevsek[*,1], Florian Caiment[*,2], Ramona Nudischer[*,3], Hans Gmuender[*,4], Irina Agarkova[11], Francis L Atkinson[12], Ivo Bachmann[9], Vanessa Baier[6], Gal Barel[5], Chris Bauer[9], Stefan Boerno[10], Nicolas Bosc[12], Olivia Clayton[3], Henrik Cordes[6], Sally Deeb[4], Stefano Gotta[4], Patrick Guye[11], Anne Hersey[12], Fiona M I Hunter[12], Laura Kunz[1], Alex Lewalle[7], Matthias Lienhard[5], Jort Merken[8], Jasmine Minguet[12], Bernardo Oliveira[7], Carla Pluess[3], Ugis Sarkans[12], Yannick Schrooders[2], Johannes Schuchhardt[9], Ines Smit[12], Christoph Thiel[6], Bernd Timmermann[10], Marcha Verheijen[2], Timo Wittenberger[4], Witold Wolski[1], Alexandra Zerck[9], Stephane Heymans[8], Lars Kuepfer[6], Adrian Roth[3], Ralph Schlapbach[1], Steven Niederer[7], Ralf Herwig[**,§,5], Jos Kleinjans[**,2]

[1] Functional Genomics Center, ETH Zurich, Switzerland

[2] Maastricht University, Department of Toxicogenomics, The Netherlands

[3] Roche Pharma Research and Early Development, Roche Innovation Center Basel, Basel, Switzerland

[4] Genedata AG, Basel, Switzerland

[5] Max-Planck-Institute for Molecular Genetics, Dep. Computational Molecular Biology, Berlin, Germany

[6] Institute of Applied Microbiology, RWTH Aachen, Germany

[7] King's College London, Dep. Biomedical Engineering, United Kingdom

[8] Maastricht University, CARIM School for Cardiovascular Diseases, The Netherlands.

[9] MicroDiscovery GmbH, Berlin, Germany

[10] Max-Planck-Institute for Molecular Genetics, Sequencing Unit, Berlin, Germany

[11] Insphero AG, Schlieren, Switzerland

[12] European Molecular Biology Laboratory, European Bioinformatics Institute (EMBL-EBI), Hinxton, United Kingdom

* equal contribution as first authors
** equal contribution as last authors
§ corresponding author

Supplementary Methods

1. Dosing scheme

3D cardiac microtissues were treated with four anthracyclines (DOX: doxorubicin, EPI: epirubicin, IDA: idarubicin, DAU: daunorubicin) at two different doses (THE: therapeutic dose, TOX: toxic dose). The dosing scheme is described in Supplementary Table 1.

Supplementary Table 1. Dosing scheme overview.

Time_range	DOX		EPI		IDA		DAU	
	THE [uM]	TOX [uM]	THE [uM]	TOX [uM]	THE [uM]	TOX [uM]	THE [uM]	TOX [uM]
**0 - 2 h**	0,210948	0,601646	0,220762	0,900051	0,004796	0,087217	0,033797	1,074738
**2 - 8 h**	0,016106	0,045939	0,012507	0,050995	0,001429	0,022968	0,003623	0,115513
**8 - 24 h**	0,008439	0,024081	0,004445	0,018124	0,000941	0,009668	0,000521	0,016609
24 - 26 h	0,217426	0,620128	0,223587	0,911572	0,005744	0,105634	0,034137	1,085622
26 - 32 h	0,022025	0,06284	0,01494	0,060921	0,002198	0,037454	0,00393	0,125334
32 - 48 h	0,013171	0,037611	0,006153	0,025087	0,001527	0,016465	0,000762	0,024283
48 - 50 h	0,221385	0,631448	0,224894	0,916941	0,006329	0,115424	0,034336	1,091998
50 - 56 h	0,025699	0,073365	0,016115	0,065701	0,002674	0,045688	0,004115	0,13123
**56 - 72 h**	0,016188	0,046262	0,007055	0,028763	0,001886	0,020168	0,000915	0,029173
72 - 144 h *	0,039025	0,111473	0,028365	0,115649	0,002835	0,037997	0,004697	0,149528
144 - 146 h	0,227455	0,648925	0,226499	0,923458	0,00712	0,123474	0,034704	1,103816
146 - 152 h	0,031366	0,089689	0,017592	0,071738	0,00332	0,052668	0,004467	0,142489
**152 - 168 h**	0,020879	0,059793	0,00824	0,033595	0,002372	0,023324	0,001228	0,039173
168 - 170 h	0,22794	0,650331	0,226603	0,923868	0,00717	0,123695	0,034754	1,10543
170 - 176 h	0,031819	0,091003	0,017689	0,072131	0,00336	0,052869	0,004516	0,144042
176 - 192 h	0,021255	0,06089	0,008318	0,033915	0,002403	0,023418	0,001273	0,040596
192 - 194 h	0,22826	0,651272	0,226675	0,92413	0,0072	0,123808	0,034796	1,10676
194 - 200 h	0,032122	0,09188	0,017752	0,072374	0,003386	0,05297	0,004556	0,145327
200 - 216 h	0,021506	0,061619	0,008367	0,034113	0,002422	0,023468	0,00131	0,041772
216 - 218 h	0,228477	0,651882	0,226713	0,924313	0,007219	0,123867	0,03483	1,107861
218 - 224 h	0,032321	0,092462	0,01779	0,072521	0,003401	0,053025	0,004589	0,14639
**224 - 240 h**	0,021673	0,062106	0,008398	0,034238	0,002434	0,023495	0,00134	0,042747
240 - 312 h *	0,041756	0,119399	0,02897	0,118114	0,003087	0,039272	0,00499	0,158908
312 - 314 h	0,228813	0,652888	0,226767	0,924539	0,007245	0,123939	0,034918	1,110684
314 - 320 h	0,032639	0,093402	0,017843	0,07275	0,003422	0,053089	0,004674	0,149117
**320 - 336 h**	0,021937	0,062885	0,008441	0,034417	0,00245	0,023527	0,001419	0,045249

*dose determined by averaging over exposure profile for the respective time range

For each treatment, physiologically-based pharmacokinetic models were developed and *in vivo* drug exposure was simulated in the interstitial space of the heart over two weeks of a once daily administration. Therapeutic doses were estimated from clinical dosing, whereas

toxic concentrations were computed based on *in vitro* viability experiments (IC20) in the microtissues by reverse dosimetry. From the continuous exposure profiles (Supplementary Fig. 2) an experimental setting was derived with three daily media changes for a period of 14 days (except weekends) mimicking the estimated *in vivo* PK exposure profiles. Over the weekend, spheroids were treated with an average concentration calculated from the PK exposure profile. Drugs were administered at the respective concentrations at the beginning of the each time range and material for molecular analyses was extracted at the respective end of the time range (in bold).

2. Experiments and data analyses

Effects of treatments were measured at three different molecular levels (methylome, transcriptome and proteome) and at seven time points (2h, 8h, 24h, 72h, 168h, 240h, 336h) using three replicate measurements per time point (A, B and C). Additionally, time-matched control experiments were performed (DMSO). Supplementary Table 2 lists the different experiments.

Supplementary Table 2. Overview of the molecular experiments. In red, outlier experiments are marked that didn't pass the QC procedures and were discarder in further analysis.

A. Methylation experiments*

Treatment	Dose	2h			8h			24h			72h			168h			240h			336h		
DMSO		A	B	C	A	B	C	A	B	C	A	B	C	A	B	C	A	B	C	A	B	C
DOX	Therapeutic	ABC			ABC			ABC			ABC			ABC			ABC			ABC		
DOX	Toxic	ABC			ABC			ABC			ABC			ABC			ABC			ABC		
EPI	Therapeutic	A	B	C	A	B	C	A	B	C	A	B	C	A	B	C	A	B	C	A	B	C
EPI	Toxic	A	B	C	A	B	C	A	B	C	A	B	C	A	B	C	A	B	C	A	B	C
IDA	Therapeutic	ABC			ABC			ABC			ABC			ABC			ABC			ABC		
IDA	Toxic	ABC			ABC			ABC			ABC			ABC			**ABC**			**ABC**		
DAU	Therapeutic	A	B	C	A	B	C	A	B	C	A	B	C	A	B	C	A	B	C	A	B	C
DAU	Toxic	A	B	C	A	B	C	A	B	C	A	B	C	A	B	C	A	B	C	**-**	**-**	**-**

* In the case of DOX and IDA treatments the replicate DNA samples were pooled prior to the methylation enrichment and sequencing experiments

B. Transcriptome experiments

Treatment	Dose	2h			8h			24h			72h			168h			240h			336h		
DMSO		A	B	C	A	B	C	A	B	C	A	B	C	A	B	C	A	B	C	A	B	C
DOX	Therapeutic	A	B	C	A	B	C	A	B	C	A	B	C	A	B	C	A	B	C	A	B	C
DOX	Toxic	A	B	C	A	B	C	A	B	C	A	B	C	A	B	C	A	**B**	C	A	B	C
EPI	Therapeutic	A	B	C	A	B	C	A	B	C	A	B	C	A	B	C	A	B	C	A	B	C
EPI	Toxic	A	B	C	A	**B**	C	A	B	C	A	B	C	A	B	C	A	B	C	A	B	C
IDA	Therapeutic	A	B	C	A	B	C	A	B	C	A	B	C	A	B	C	A	B	**C**	A	B	C
IDA	Toxic	A	B	C	A	B	C	A	B	C	A	B	C	A	B	C	A	B	C	A	**B**	C
DAU	Therapeutic	A	B	C	A	B	C	A	B	C	A	B	C	A	B	C	A	B	C	A	B	C
DAU	Toxic	A	B	C	A	B	C	A	B	C	A	B	C	A	B	**C**	A	B	C	**-**	**-**	**-**

**C. Proteome experiments**																						
Treatment	Dose	2h			8h			24h			72h			168h			240h			336h		
DMSO		A	B	C	A	B	C	A	B	C	A	B	C	A	B	C	A	B	C	A	B	C
DOX	Therapeutic	A	B	C	A	B	C	A	B	C	A	B	C	A	B	C	A	B	C	A	B	C
DOX	Toxic	A	B	C	A	B	C	A	B	C	A	B	C	A	B	C	A	B	C	A	B	C
EPI	Therapeutic	A	B	C	A	B	C	A	B	C	A	B	C	A	B	C	A	B	C	A	B	C
EPI	Toxic	A	B	C	A	B	C	A	B	C	A	B	C	A	B	C	A	B	C	A	B	C
IDA	Therapeutic	A	B	C	A	B	C	A	B	C	A	B	C	A	B	C	A	B	C	A	B	C
IDA	Toxic	A	B	C	A	B	C	A	B	C	A	B	C	A	B	C	**A**	**B**	**C**	**A**	**B**	**C**
DAU	Therapeutic	A	B	C	A	B	C	A	B	C	A	B	C	A	B	C	A	B	C	A	B	C
DAU	Toxic	A	B	C	A	B	C	A	B	C	A	B	C	A	B	C	A	B	C	-	-	-

Different kinds of analysis were performed with the molecular data:

Longitudinal data analysis: This analysis was performed with the *transcriptome* and *proteome* data with the goal to identify response genes/proteins that have an expression profile over time that is different from the control experiments. This analysis was done using the R/Bioconductor package MaSigPro and applying a two-step polynomial regression model with maximal degree of 2 [1]. For each treatment and dose the respective 21 experiments (7 time points x 3 replicates) along with the 21 control experiments were summarized into the polynomial model and significant deviations were identified according to the respective P-values (Supplementary Fig. 6 and Methods).

Time-point specific data analysis: This analysis was performed with the *transcriptome* and *proteome* data with the goal to identify differentially expressed genes/proteins at single time points comparing the replicates per time point (3x treatment vs 3x controls) with a statistical test (in the case of transcriptome data with DESeq2, in the case of proteome data with Student’s t-test; cf. Methods).

Pooled time point analysis: This analysis was performed with the methylation data in order to identify differentially methylated regions (DMRs) between AC treatment and controls across the seven time points, with prior averaging of the replicates per time point, using the QSEA tool (7x treatment vs 7x controls). QSEA transforms the MeDIP-seq enrichment counts for each genomic region into a bisulfite-like % methylation value using a Bayesian model. The statistical analysis for identifying DMRs is then based on a generalized linear model [2].

3. Basic data statistics

3.1 Methylome analysis

QC was performed based on the number of paired-end reads mapped to the reference genome, the coverage of the genome sequence and follow-up visual inspection of all experiments per treatment group using PCA. This excluded 2 out of 130 (1.5%) experiments (IDA, toxic dose, 240h, and IDA, toxic dose, 336h; Supplementary Table 2A). In both cases less than 5 Mio reads were available and the experiments were discarded from further analysis.

For each treatment and dose differentially methylated regions (DMRs) were identified using pooled time point analysis comparing the treatment samples (7; for IDA, toxic only 5) against the control samples (7) using QSEA [2]. This led to the following results:

Comparison	Number of regions tested	Number of DMRs
DOX vs DMSO therapeutic dose	6,965,162	45,440
DOX vs DMSO toxic dose	6,646,623	96,365
EPI vs DMSO therapeutic dose	8,018,940	158,762
EPI vs DMSO toxic dose	7,824,113	55,141
IDA vs DMSO therapeutic dose	6,808,151	61,453
IDA vs DMSO toxic dose	6,492,202	36,060
DAU vs DMSO therapeutic dose	9,416,171	174,893
DAU vs DMSO toxic dose	8,999,573	36,349

3.2 Transcriptome analysis

First, experiments with an insufficient number of mapped paired reads were discarded. Additionally, we applied visual inspection using PCA, heatmaps based on expressed genes and Cook’s distance measures. Where these measures gave consistent negative results we flagged the experiments as ‘outliers’ and excluded them from further analyses. In total 5 out of 186 (2.7%) experiments were discarded (Supplementary Table 2B).

Sequences were mapped to the following genomic features:

**Total number of genes:**	**58,219**
Protein-coding genes:	19,817
Long non-coding RNAs:	15,787
Small non-coding RNAs:	7,568
Pseudogenes:	14,637
Immunoglobulin/T-cell receptor gene segments:	410

After mapping, quantification and normalization of the gene expression (Methods) we performed longitudinal data analysis using the polynomial regression model in order to identify dynamic response genes. The number of dynamic response genes identified by the polynomial regression model are:

Comparison	Number of dynamic response genes
DOX vs DMSO therapeutic dose	88
DOX vs DMSO toxic dose	116
EPI vs DMSO therapeutic dose	127
EPI vs DMSO toxic dose	158
IDA vs DMSO therapeutic dose	138
IDA vs DMSO toxic dose	250
DAU vs DMSO therapeutic dose	366
DAU vs DMSO toxic dose	116

Additionally, we performed time-point specific data analysis comparing for each treatment, dose and time-point the gene expression measured on the three replicate samples against the three replicates of the time-matched control samples. We used DESeq2 for identifying differentially expressed genes and corrected the p-values with the Benjamini-Hochberg method using a threshold of q < 0.05. This led to the following number of differentially expressed genes:

Experiment (Treatment_Dose_TimePoint)	DEseq2 Treatment vs. DMSO (BH q < 0.05)
DAU_Ther_002	934
DAU_Ther_008	1292
DAU_Ther_024	745
DAU_Ther_072	970
DAU_Ther_168	1108
DAU_Ther_240	987
DAU_Ther_336	593
DAU_Tox_002	113
DAU_Tox_008	242
DAU_Tox_024	271
DAU_Tox_072	532
DAU_Tox_168	1362
DAU_Tox_240	1242
DAU_Tox_336	not performed
DOX_Ther_002	639
DOX_Ther_008	667
DOX_Ther_024	455
DOX_Ther_072	747
DOX_Ther_168	2166
DOX_Ther_240	1004
DOX_Ther_336	1819
DOX_Tox_002	726
DOX_Tox_008	601
DOX_Tox_024	520
DOX_Tox_072	1067
DOX_Tox_168	2394
DOX_Tox_240	2535
DOX_Tox_336	2748
EPI_Ther_002	1584
EPI_Ther_008	1293
EPI_Ther_024	1196
EPI_Ther_072	1567

EPI_Ther_168	1759
EPI_Ther_240	966
EPI_Ther_336	1886
EPI_Tox_002	862
EPI_Tox_008	551
EPI_Tox_024	2345
EPI_Tox_072	1165
EPI_Tox_168	1705
EPI_Tox_240	3056
EPI_Tox_336	2111
IDA_Ther_002	1850
IDA_Ther_008	1687
IDA_Ther_024	1518
IDA_Ther_072	1485
IDA_Ther_168	1802
IDA_Ther_240	1127
IDA_Ther_336	1347
IDA_Tox_002	960
IDA_Tox_008	1442
IDA_Tox_024	844
IDA_Tox_072	687
IDA_Tox_168	1250
IDA_Tox_240	3883
IDA_Tox_336	2422

3.3. Proteome analysis

QC was performed on visual inspection using PCA. This excluded 6 out of 186 (3.2%) experiments (Supplementary Table 2C).
Proteins were detected after m/z alignment, RT alignment, peak detection, isotope clustering and identification and validation of peptides (Methods).

The following numbers of proteins were detected in the samples and were identified as dynamic response proteins with the polynomial regression model:

Experiment (Treatment_Dose_TimePoint)	Quantified proteins	Number of dynamic response proteins
DOX_THE_002	1238	
DOX_THE_008	1226	
DOX_THE_024	1205	
DOX_THE_072	1249	
DOX_THE_168	1154	
DOX_THE_240	1136	
DOX_THE_336	1167	270
DOX_TOX_002	980	
DOX_TOX_008	972	
DOX_TOX_024	959	
DOX_TOX_072	924	
DOX_TOX_168	819	
DOX_TOX_240	791	
DOX_TOX_336	690	242
EPI_THE_002	932	210

EPI_THE_008		923	
EPI_THE_024		924	
EPI_THE_072		906	
EPI_THE_168		943	
EPI_THE_240		883	
EPI_THE_336		931	
EPI_TOX_002		942	
EPI_TOX_008		857	
EPI_TOX_024		796	
EPI_TOX_072		773	
EPI_TOX_168		795	
EPI_TOX_240		510	
EPI_TOX_336		559	226
IDA_THE_002		648	
IDA_THE_008		607	
IDA_THE_024		649	
IDA_THE_072		678	
IDA_THE_168		588	
IDA_THE_240		635	
IDA_THE_336		625	176
IDA_TOX_002		681	
IDA_TOX_008		591	
IDA_TOX_024		641	
IDA_TOX_072		669	
IDA_TOX_168		473	
IDA_TOX_240	discarded		
IDA_TOX_336	discarded		157
DAU_THE_002		790	
DAU_THE_008		864	
DAU_THE_024		871	
DAU_THE_072		875	
DAU_THE_168		863	
DAU_THE_240		891	
DAU_THE_336		949	158
DAU_TOX_002		813	
DAU_TOX_008		699	
DAU_TOX_024		752	
DAU_TOX_072		737	
DAU_TOX_168		690	
DAU_TOX_240		314	
DAU_TOX_336	not performed		120
DMSO_002		1317	
DMSO_008		1390	
DMSO_024		1340	
DMSO_072		1207	
DMSO_168		1344	
DMSO_240		1340	
DMSO_336		1304	

4. ACT response network construction

We used the PPI network provided by ConsensusPathDB [3,4] as a scaffold and performed network propagation with the weights derived from the proteome and transcriptome experimental data. The purpose of this analysis was to derive an ACT response network describing the most common *in vitro* responses to the four anthracyclines. This ACT response network (Fig. 4) was generated by the following procedure:

1. For each treatment and dose map the dynamic response proteins (Supplementary Data 2) to the PPI network and initialize the corresponding nodes with the P-value scores that describe the significance of the deviation of the treatment temporal profile from the control temporal profile according to Supplementary Fig. 11. Numbers of initialized proteins and score densities are shown in the figure. Perform network propagation of the initialized weights using random walk with restart [5] and extract the final computed network module (genes for the respective modules are shown in Supplementary Data 4). This yields 8 different computed network modules for the proteome data (4 treatments x 2 doses).
2. For each treatment and dose map the dynamic response genes (Supplementary Data 3) to the PPI network and initialize the corresponding nodes with the P-value scores that describe the significance of the deviation of the treatment temporal profile from the control temporal profile according to Supplementary Fig. 11. Numbers of initialized genes and score densities are shown in the figure. Perform network propagation of the initialized weights using random walk with restart [5] and extract the final computed network module (genes for the respective modules are shown in Supplementary Data 4). This yields 8 different computed network modules for the transcriptome data (4 treatments x 2 doses).
3. For each treatment and dose map the dynamic response proteins and transcripts to the PPI network and initialize the corresponding nodes with the joined P-value scores according to Supplementary Fig. 11. Numbers of initialized proteins/genes and score densities are shown in the figure. Perform network propagation and extract the final computed network module (genes for the respective modules are shown in Supplementary Data 4). This yields 8 different computed network modules for the integrated data (4 treatments x 2 doses).

As an intermediate result we observed that the final modules derived from the integrated data (Supplementary Fig. 12-13) are larger in size (Supplementary Fig. 14) and contain more functional information (Supplementary Fig. 15) than the modules derived from proteome data only and transcriptome data only. Thus, this approach demonstrates an efficient data integration strategy that can easily be extrapolated with further data sets.

We thus continued with the four network modules derived from integrated data (Supplementary Data 4), and we constructed the ACT response networks for therapeutic and toxic doses respectively from the four individual AC networks computed in step 3 above for the respective dose. Since we were interested in *common* responses at the two doses we overlaid the modules for the four treatments and kept only those proteins and their interactions that were present in at least 2 of the 4 treatments. We were then particularly interested in the effects of the lower therapeutic dose because it is pharmacologically relevant and adapted to the clinical dosing in patients. At therapeutic dose this left us with 175 proteins and their interactions and this network is displayed and discussed in Fig. 4 and further evaluated for disease content with biopsies and physiological modelling.

Supplementary Figures

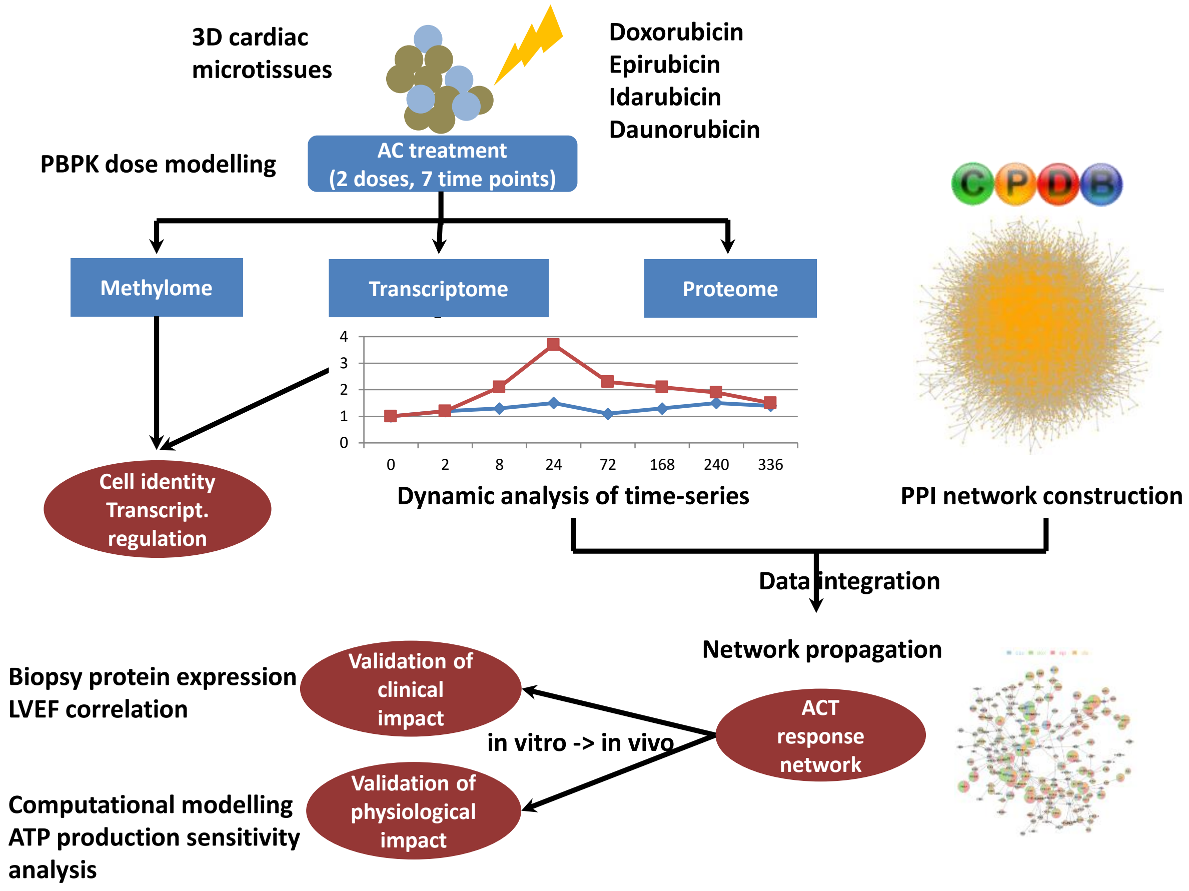

Supplementary Figure 1 Study schema. 3D cardiac microtissues are grown and treated over a time period of 14 days with four anthracycline drugs. PBPK modelling is used to infer *in vivo* drug exposure levels at two doses (therapeutic and toxic doses). Omics measurements were done at seven different time points (2h, 8h, 24h, 72h, 168h, 240h, 336h). Methylome measurements were used to characterize cell identity of cardiac microtissues and to infer effects of AC treatment on transcriptional regulation. Proteome and transcriptome measurements were used to characterize dynamic cellular responses. Time series data was mapped to a large PPI network in order to integrate the data and to identify a common ACT response network across the treatments. The *in vitro* inferred ACT response network was then tested for clinical relevance in cardiomyopathy patient biopsies and for physiological relevance on ATP production with sensitivity analysis using an established computational model of the human mitochondrion.

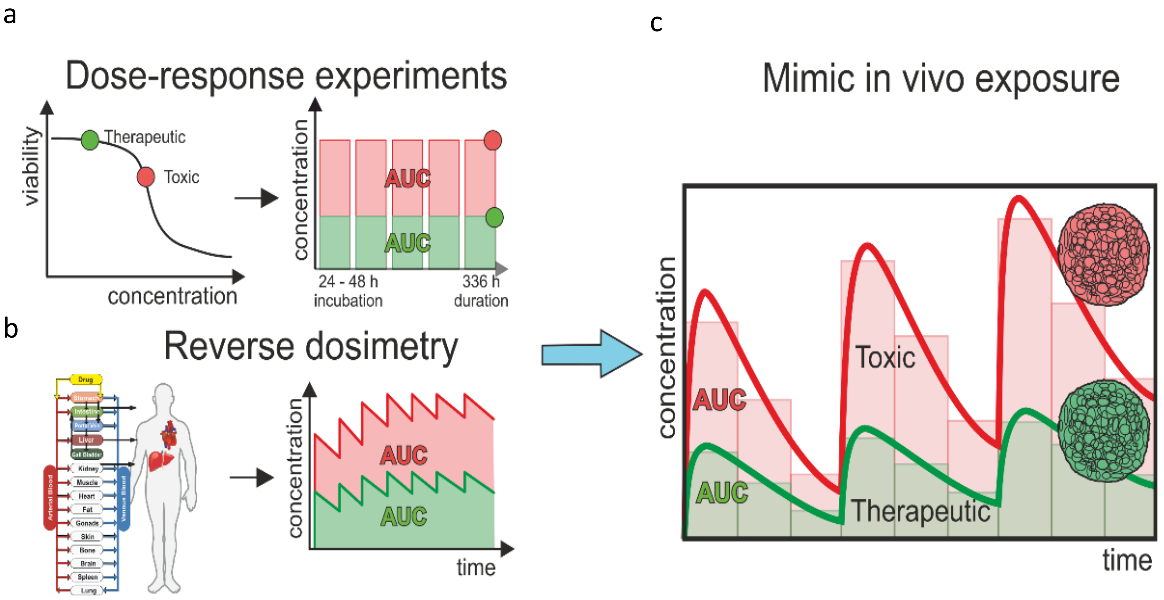

Supplementary Figure 2 ***In vitro*** **assay dosing.** Assay set up with PBPK models and reverse dosimetry. The *in vitro* assay design aims to mimic organ-specific *in vivo* drug exposure. (a) Dose-response experiments for the *in vitro* cell system are conducted to identify a toxic exposure resulting in a reduction of 20 % cell viability. (b) PBPK models are used to estimate the anticipated *in vivo* dose that is needed in a once daily dosing regimen to result in the same exposure via reverse dosimetry. (c) The continuous drug exposure profiles are translated into an experimental setting with three daily media changes mimicking the estimated *in vivo* PK exposure profiles (cf. Supplementary Methods).

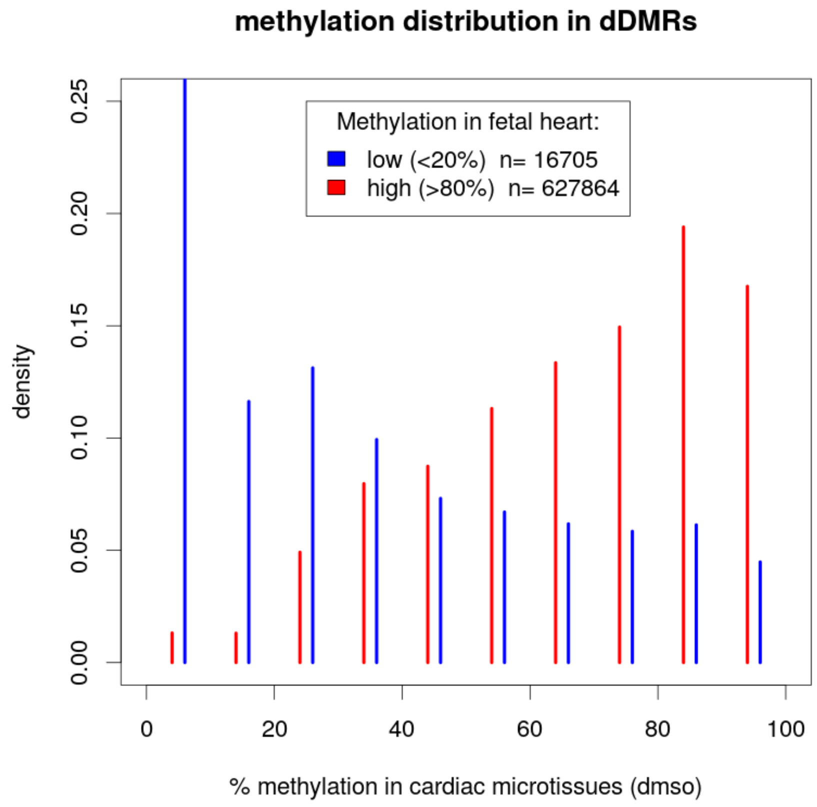

Supplementary Figure 3 Heart-specific dynamic differentially methylated regions (dDMRs) in 3D cardiac microtissues. Ziller et al. [6] have performed whole-genome bisulfite sequencing of 30 human cell lines of different cell types and developmental stages and identified tissue-specific dynamic methylated regions (dDMRs). The dynamic methylome leads to the identification of genomic regions that are differentially methylated between fetal heart and other cell lines. We selected the lowly (<20%: 16,705 dDMRs) and highly (>80%: 627,864 dDMRs) methylated dDMRs from that study and compared their methylation status in the IPSC-derived cardiac microtissues (control samples). Blue curve: distribution of lowly methylated dDMRs in cardiac microtissues; red curve: distribution of highly methylated dDMRs in cardiac microtissues.

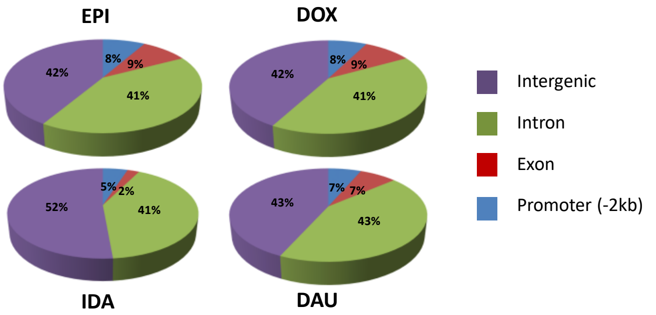

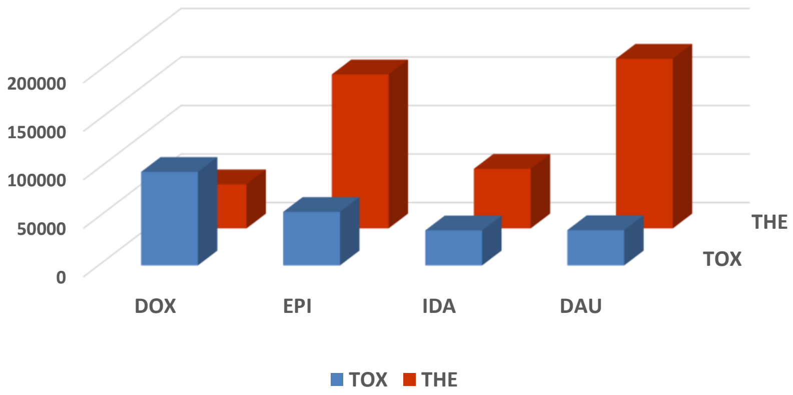

Supplementary Figure 4 Differentially methylated regions in cardiac microtissues. Genomic annotation of differentially methylated regions and overall statistics for the different experimental conditions. AC-induced methylation values were compared against DMSO controls in order to identify differentially methylated regions (DMRs) using the QSEA tool (Lienhard et al., 2017): 42-52% of the DMRs fall in intergenic regions. Genic DMRs are mostly in introns (41-43%) with only a minority of 2-9% of the DMRs corresponding to exon and 5-8% corresponding to promoter sequences (-2kb upstream of the transcription start site). ACs show large differences in methylation effects: DAU and EPI induced 3-5 times more changes in methylation than IDA and DOX at therapeutic dose whereas overall effects are generally lower (except for DOX) at toxic doses.

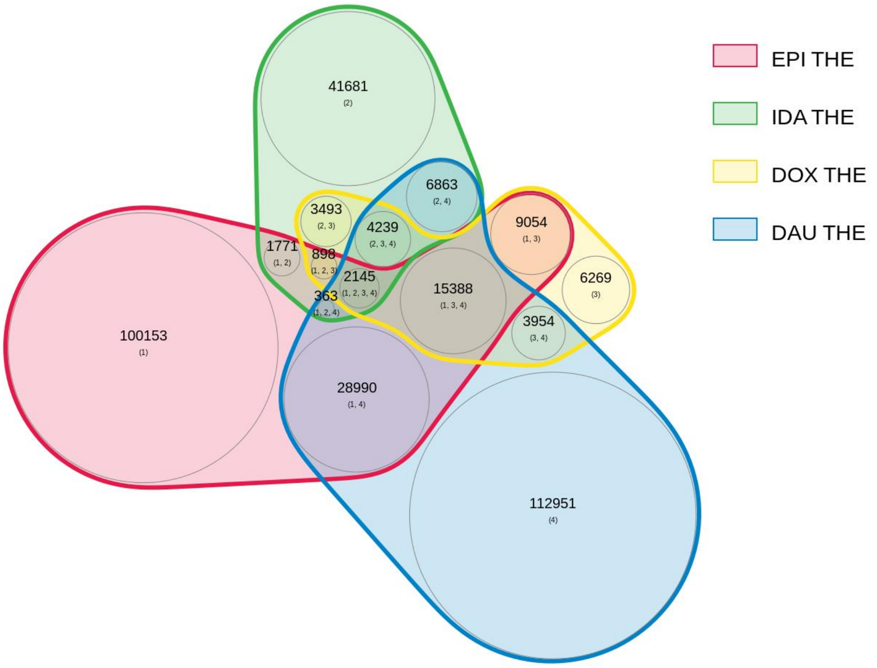

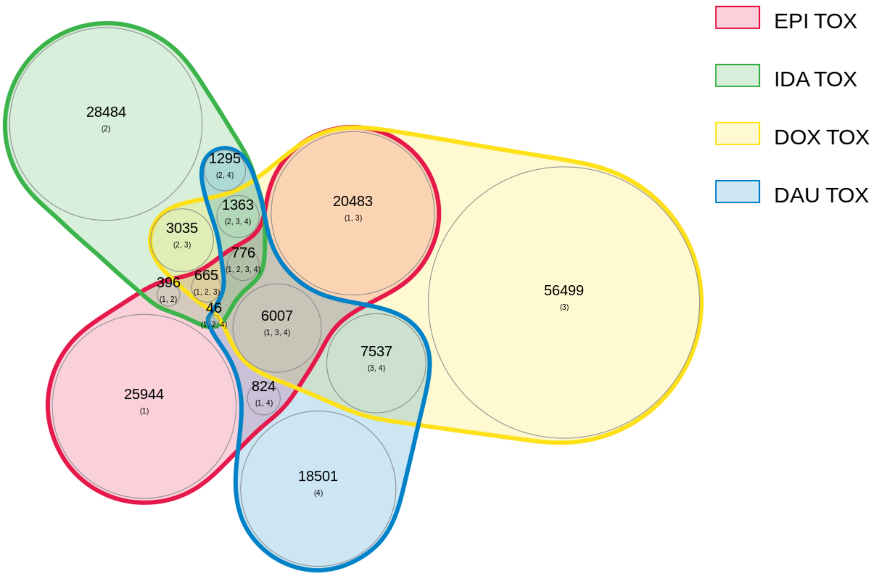

Supplementary Figure 5 Differentially methylated regions in cardiac microtissues. VENN diagrams of DMRs from the different treatments. Top: therapeutic dose. Bottom: toxic dose.

a

AC vs Control design matrix

	Time	Replicate	Control	Treatment
C_2h_1	2	1	1	0
C_2h_2	2	1	1	0
C_2h_3	2	1	1	0
C_8h_1	8	2	1	0
C_8h_2	8	2	1	0
C_8h_3	8	2	1	0
C_24h_1	24	3	1	0
C_24h_2	24	3	1	0
C_24h_3	24	3	1	0
C_72h_1	72	4	1	0
C_72h_2	72	4	1	0
C_72h_3	72	4	1	0
C_168h_1	168	5	1	0
C_168h_2	168	5	1	0
C_168h_3	168	5	1	0
C_240h_1	240	6	1	0
C_240h_2	240	6	1	0
C_240h_3	240	6	1	0
C_336h_1	336	7	1	0
C_336h_2	336	7	1	0
C_336h_3	336	7	1	0
Treatment_2h_1	2	8	0	1
Treatment_2h_2	2	8	0	1
Treatment_2h_3	2	8	0	1
Treatment_8h_1	8	9	0	1
Treatment_8h_2	8	9	0	1
Treatment_8h_3	8	9	0	1
Treatment_24h_1	24	10	0	1
Treatment_24h_2	24	10	0	1
Treatment_24h_3	24	10	0	1
Treatment_72h_1	72	11	0	1
Treatment_72h_2	72	11	0	1
Treatment_72h_3	72	11	0	1
Treatment_168h_1	168	12	0	1
Treatment_168h_2	168	12	0	1
Treatment_168h_3	168	12	0	1
Treatment_240h_1	240	13	0	1
Treatment_240h_2	240	13	0	1
Treatment_240h_3	240	13	0	1
Treatment_336h_1	336	14	0	1
Treatment_336h_2	336	14	0	1
Treatment_336h_3	336	14	0	1

b

Polynomial regression model (degree ≤ 2)

$$y_{ijr} = (\beta_{0,C} + \beta_{0,TvsC}) + (\beta_{1,C} + \beta_{1,TvsC})\, t_{ijr} + (\beta_{2,C} + \beta_{2,TvsC})\, t_{ijr}^2 + \varepsilon_{ijr}$$

Regression coefficients for the control group

Regression coefficients for the specific differences between the treatment and the control groups

i: treatment or control (i=1,2)
j: time point (j=1,…,7)
r: replicate (r=1,2,3)

c

Linear and quadratic treatment effect

	$\beta_{0,C}$	$\beta_{0,TvsC}$	$\beta_{1,C}$	$\beta_{1,TvsC}$	$\beta_{2,C}$	$\beta_{2,TvsC}$
**Beta**	10.55	1.01	0.00	0.03	0.00	-0.01
**P-value**	1.25E-45	3.02E-05	NA	1.68E-05	NA	6.63E-07

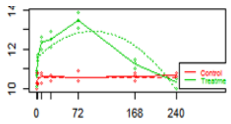

Linear treatment effect

	$\beta_{0,C}$	$\beta_{0,TvsC}$	$\beta_{1,C}$	$\beta_{1,TvsC}$	$\beta_{2,C}$	$\beta_{2,TvsC}$
**Beta**	10.45	0.00	0.00	-0.03	0.00	0.00
**P-value**	3.12E-59	NA	NA	3.89E-31	NA	NA

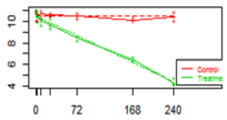

Constant (batch) effect

	$\beta_{0,C}$	$\beta_{0,TvsC}$	$\beta_{1,C}$	$\beta_{1,TvsC}$	$\beta_{2,C}$	$\beta_{2,TvsC}$
**Beta**	10.50	4.98	0.00	0.00	0.00	0.00
**P-value**	4.95E-56	3.70E-37	NA	NA	NA	NA

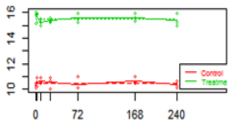

Supplementary Figure 6 Longitudinal data analysis using polynomial regression. (**a**) Design of experiments. Every AC treatment at a given dose is measured at seven time points with time-matched controls with three replicates giving rise to 42 different experiments. (**b**) Polynomial regression model (degree ≤ 2). A two-step procedure is carried out using MaSigPro [1]. (**c**) Output. Fitted regression coefficients and corresponding P-values. Dynamic response proteins/genes are then selected based on the regression coefficients $\beta_{0,TvsC}, \beta_{1,TvsC}, \beta_{2,TvsC}$ that describe the differences between control and the treatment group with respect to the constant, linear and quadratic terms respectively (upper and middle panel). Proteins/genes with only constant effects (lower panel) are discarded since they likely are influenced by batch effects. Plots show the fitted regression curves for the treatment (green) and control (red) with dotted lines and additionally a solid line connecting the median values of the three replicates per time point.

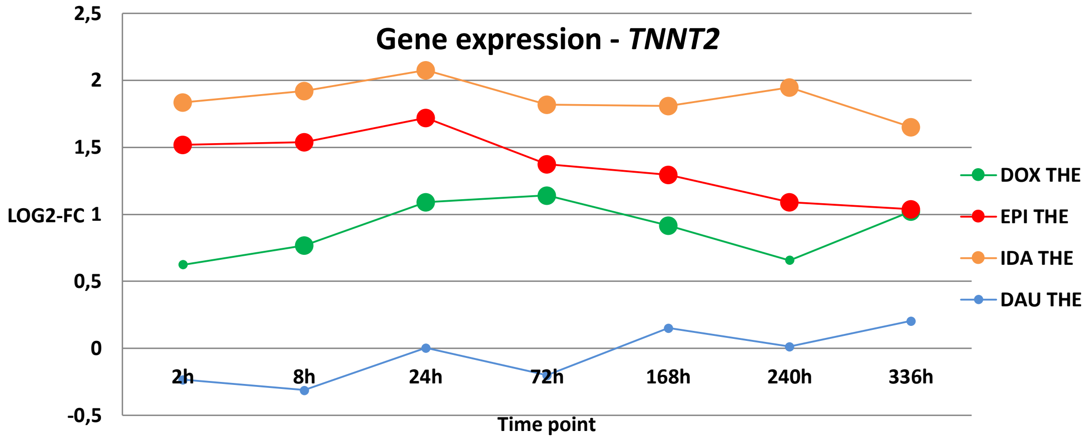

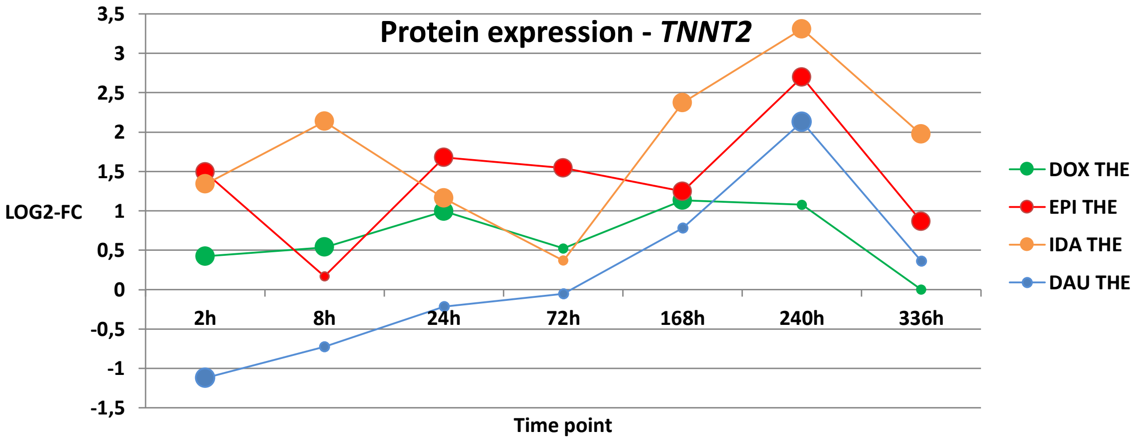

Supplementary Figure 7 Protein and gene expression of cardiac troponin T. Time-point specific ratios of TNNT2 protein and gene expression compared to controls. Y-axes show log2 fold-changes of AC treated and control experiments. X-axes show time points. Replicate values per time point were averaged. Large circles correspond to individual time points with significant deviation of treatment vs. Controls (Q<0.05). For proteome data Student's t-test was used comparing the time-point specific replicates, for transcriptome data DEseq2 was used.

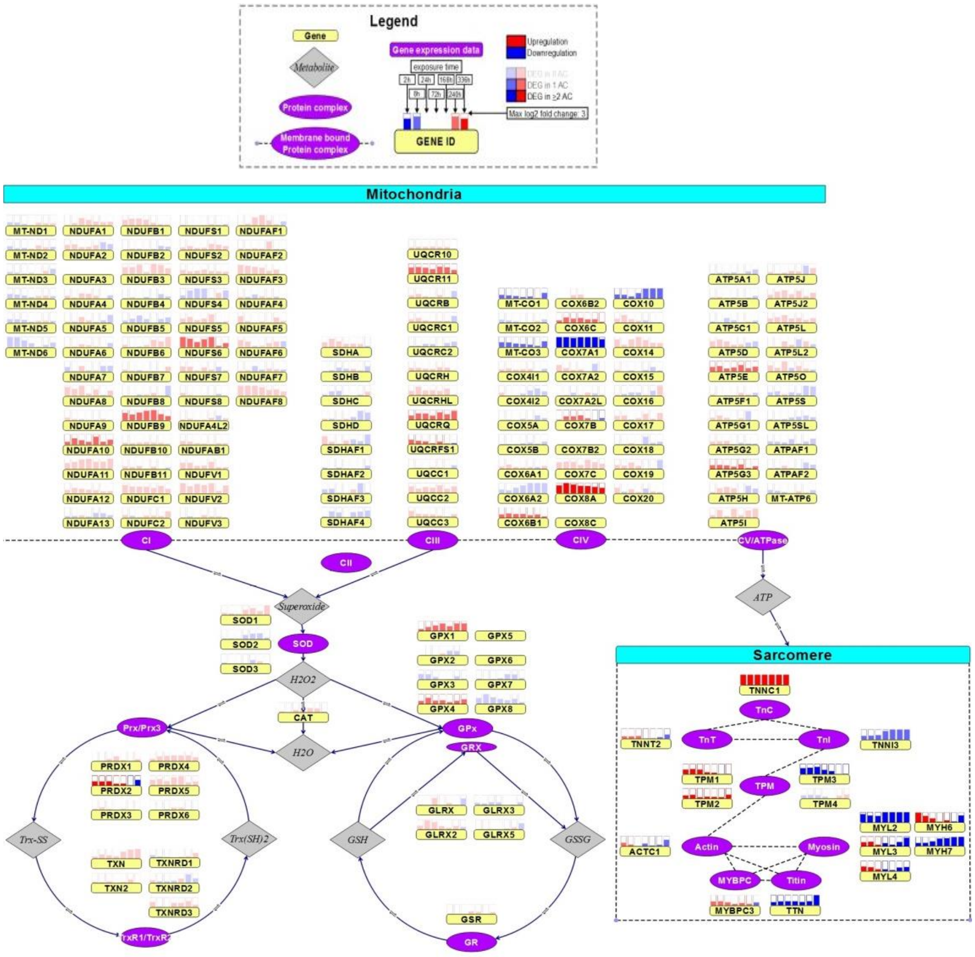

Supplementary Figure 8 Visualization of mitochondrial and sarcomeric gene expression changes after AC exposure. Exposure to the AC toxic dosing profile. Protein complexes are represented by purple ovals with the genes encoding for subunits displayed next it in yellow rectangles. On top of each gene, the boxes display the expression change over time, where each box corresponds to a specific time point. The fill level of these boxes display the log2 fold change (completely filled boxes: log2 fold change $\leq$ 3), upregulations are depicted in red and downregulations in blue. Significance, determined by MagSigPro analysis, is indicated by transparency, where the lightest genes were not DEG in any AC, medium transparent are DEG in only 1 AC and not transparent are DEG in at least 2 ACs (figure adapted from [7]).

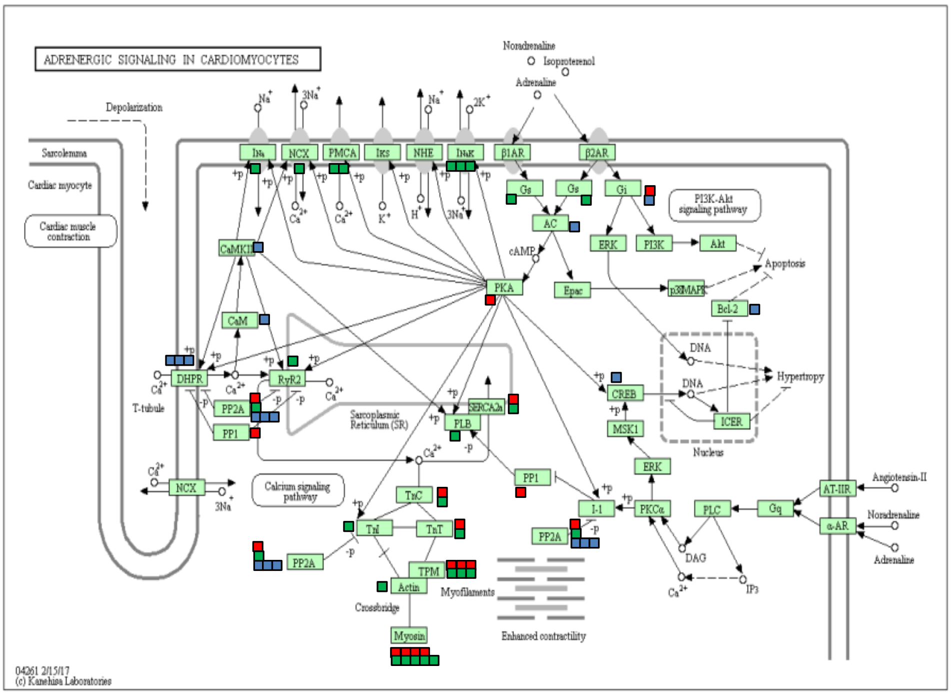

- Proteome (red): 14 genes
- Transcriptome (green): 24 genes
- Methylome (blue): 13 genes

Supplementary Figure 9. Common pathway response of response DMRs, proteins and genes with respect to the KEGG pathway *„adrenergic signalling in cardiomyocytes“*. Pathway components that were responding to one or more platforms are highlighted by colored boxes next to the component name. Multiple boxes indicate assignment of different genes to the model components. Red: proteins, green: genes, blue: DMRs.

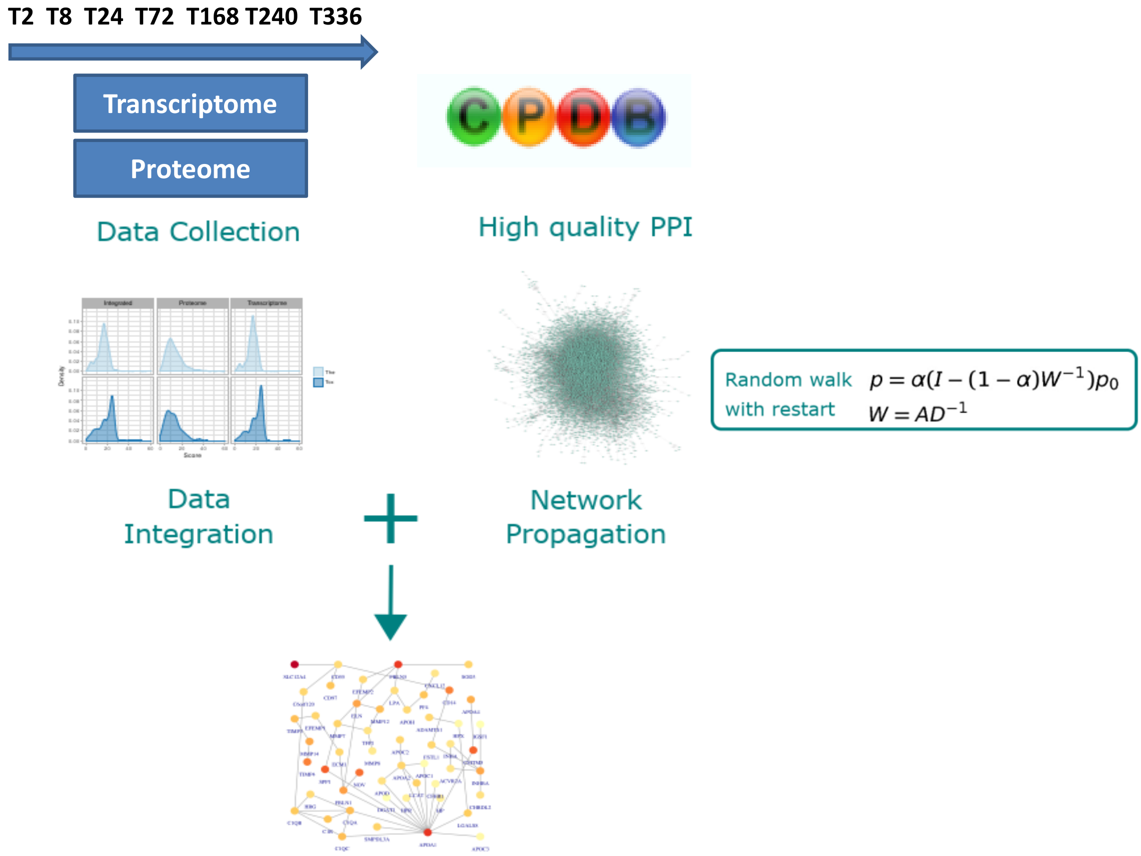

Supplementary Figure 10 Principle of network propagation concept. A large protein-protein interaction network (PPI) from 19 different data resources was agglomerated from the ConsensusPathDB molecular interaction resource consisting of 10,707 proteins and 114,516 interactions. In order to weight the proteins according to their information content with respect to time-sensitive AC treatment responses we used scores that reflect the dynamic changes of the proteins after AC treatment (Supplementary Fig. 11). If the proteins in the network show no significant dynamic response they are scored with zero. These initial weights for all proteins in the network are summarized to the initial node weight vector, p_0. Network propagation then diffuses these weights using a random walk with restart procedure and computes a final weight vector p which then allows a final reranking of all proteins along with the computation of a subnetwork containing the major connected components of the drug response. Network propagation was performed with Hotnet2 [5].

Model: $y_{ijr} = (\beta_{0,C} + \beta_{0,TvsC}) + (\beta_{1,C} + \beta_{1,TvsC})\, t_{ijr} + (\beta_{2,C} + \beta_{2,TvsC})\, t_{ijr}^2 + \varepsilon_{ijr}$

P-value: $p_{0,TvsC}$ $p_{1,TvsC}$ $p_{2,TvsC}$

i: Treatment (i=1,2)
j: Time point (j=1,..,7)
r: Replicate (r=1,2,3)

Transcriptome scoring **Proteome scoring** **Integrated scoring**

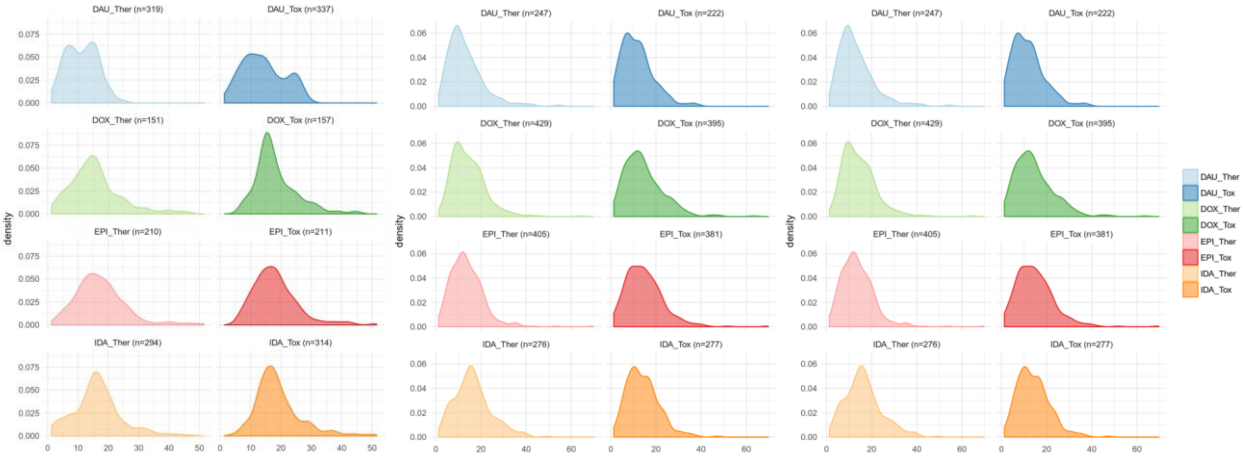

$$S_{k,T} = \sum_{l=0}^{2} -log_{10}(p_{l,TvsC}) \qquad S_{k,P} = \sum_{l=0}^{2} -log_{10}(p_{l,TvsC}) \qquad S_k = S_{k,T} + S_{k,P}$$

Supplementary Figure 11 Scoring of network nodes. Left panel: Initial node score distribution for the ACs at the different doses for transcriptome data. For each gene/protein, *k*, the score is computed as the sum of the negative log-values of the p-values for the regression coefficients in the respective polynomial model. Middle panel: Initial node score distribution for the ACs at the different doses for proteome data. Right panel: Initial node scoring distribution for the integrated approach. The integrated score is derived by summing over the scores derived from the individual experimental platforms.

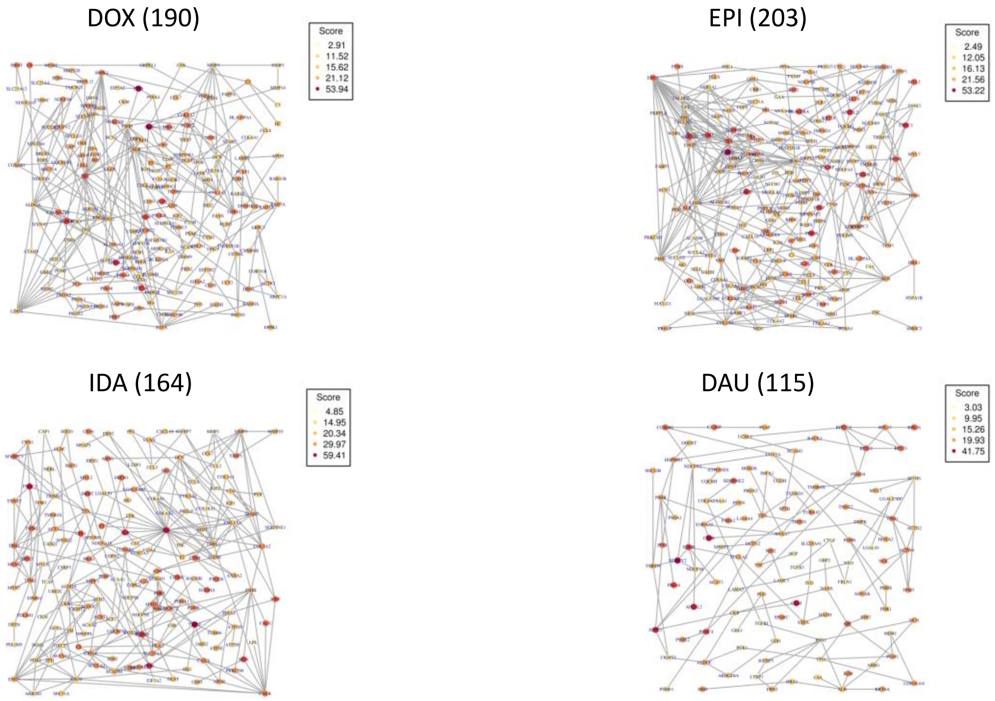

Supplementary Figure 12 AC-network modules at therapeutic doses. Largest network modules computed from integrated proteome and transcriptome data with insulated heat diffusion for ACs at therapeutic dose. Nodes were initialized based on the significance of the dynamic changes of respective AC treatment compared to DMSO control time series. Node color reflects the agglomerated heat (score) after the propagation process.

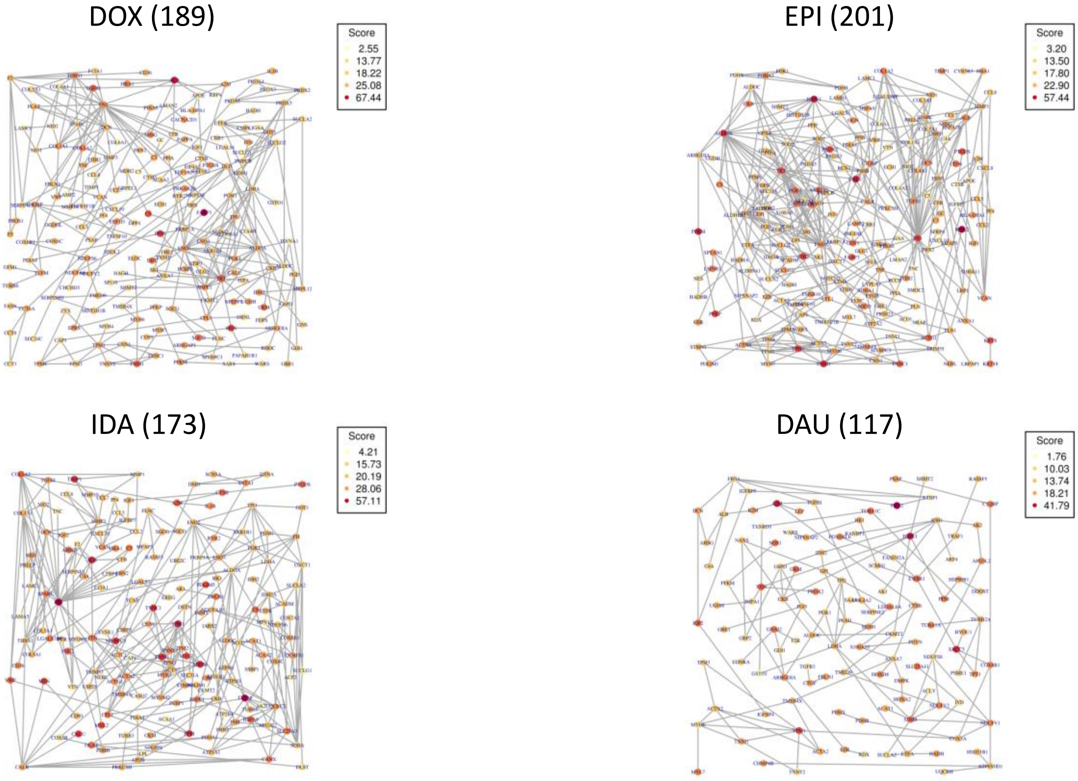

Supplementary Figure 13 AC-network modules at toxic doses. Largest network modules computed from integrated proteome and transcriptome data with insulated heat diffusion for ACs at toxic dose. Nodes were initialized based on the significance of the dynamic changes of respective AC treatment compared to DMSO control time series. Node color reflects the agglomerated heat (score) after the propagation process.

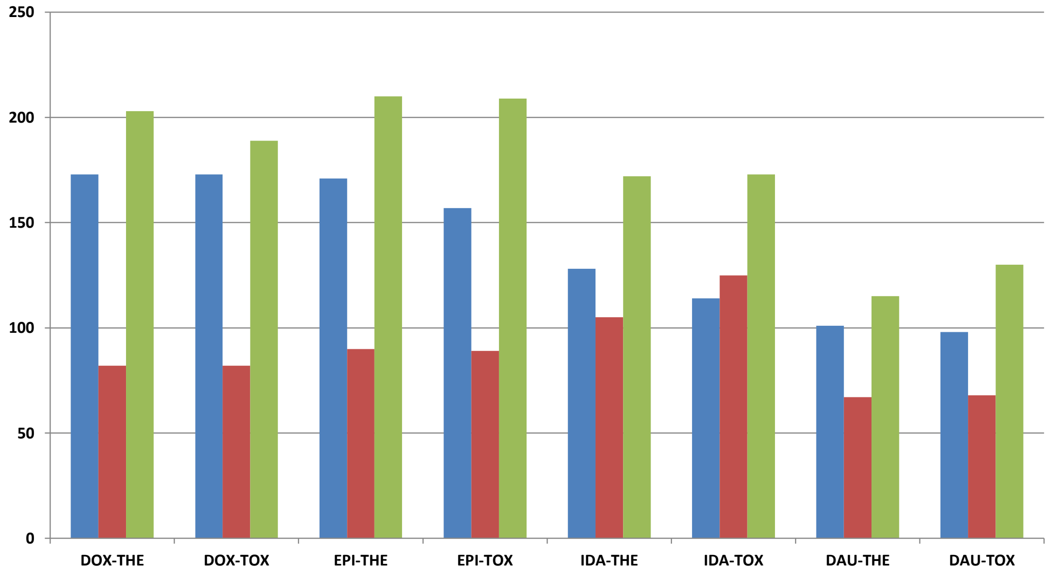

Supplementary Figure 14 Network module sizes. Sizes of all drug response networks computed from protein data (blue) and transcriptome data (red) alone and from integrated data (green).

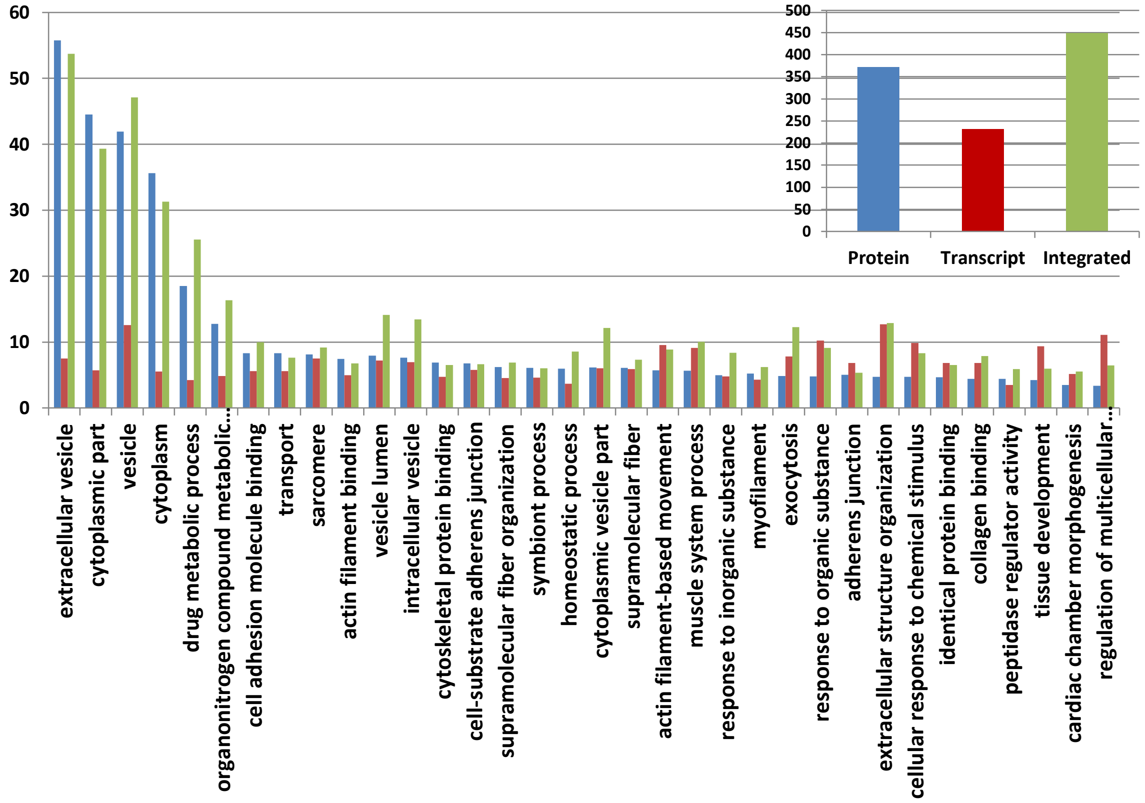

Supplementary Figure 15 Functional content of computed network modules. Enrichment of functional information for GO categories that are commonly enriched with network modules computed from protein data (blue), transcriptome data (red) and integrated data (green). X-axis displays the GO terms, Y-axis shows the enrichment score, $E_i = -\log_{10} Q_i$, for each GO term *i*, where Q_i is the Q-value of the enrichment computed with Fisher's exact test. The upper right plot summarizes the enrichment scores for each data set over all GO terms.

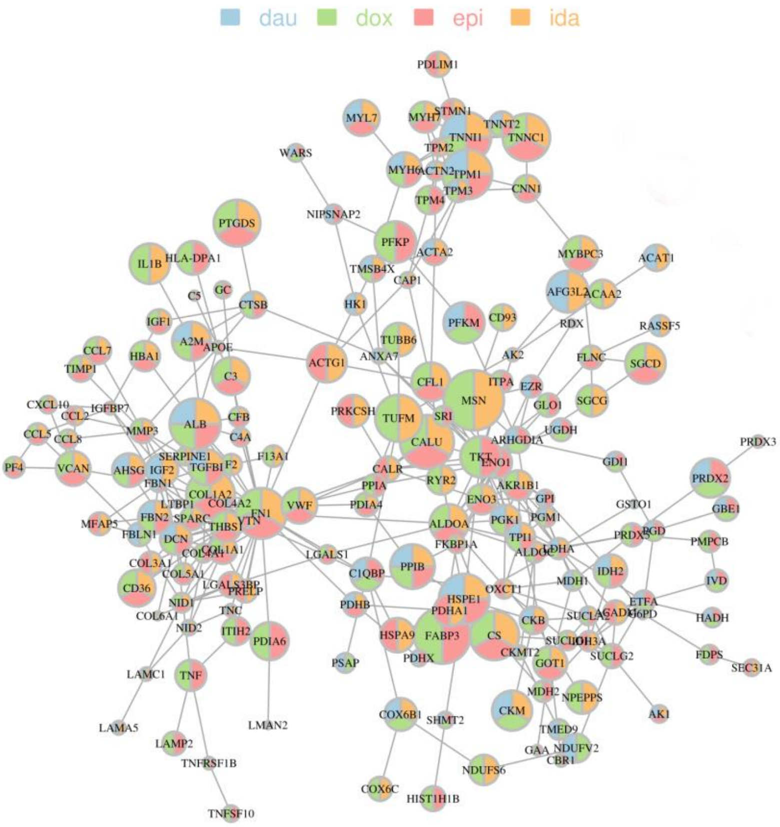

Supplementary Figure 16 Integrated ACT response network module at toxic dose. Integrated drug response network from all ACs computed from proteome and transcriptome data with insulated heat diffusion at toxic dose from a large protein-protein interaction network. Nodes were initialized based on the significance of the dynamic changes of respective AC treatment compared to DMSO control time series and for each AC a drug response network was computed (Suppl. Figs. 10,13). Nodes and their interactions that appeared in at least 2 of the individual AC networks were integrated. Node colors reflect the occurrence of the node in the individual AC networks.

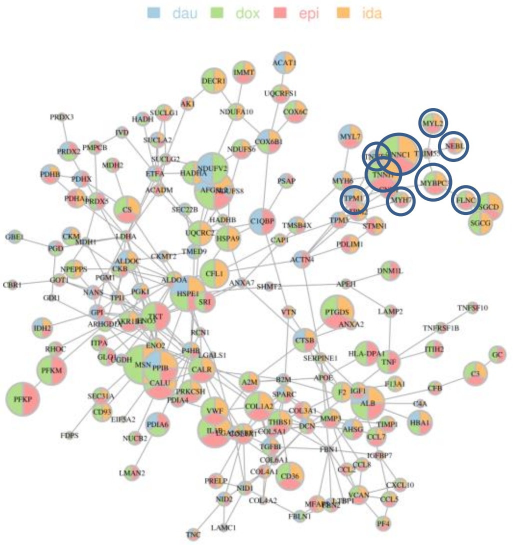

MYH7
MYL2
TNNT2
NEBL
TNNC1
FLNC
TPM1
MYBPC3

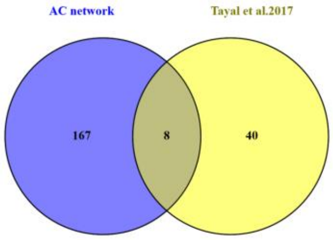

Supplementary Figure 17. 48 genes with rare and common variants in DCM and systolic heart failure identified by a recent review survey [8] overlap with the integrated response network with 8 genes (TNNT2, FLNC, MYBPC3, MYH7, MYL2, TNNC1, TPM1, NEBL) resulting in an odds ratio of 10.20.

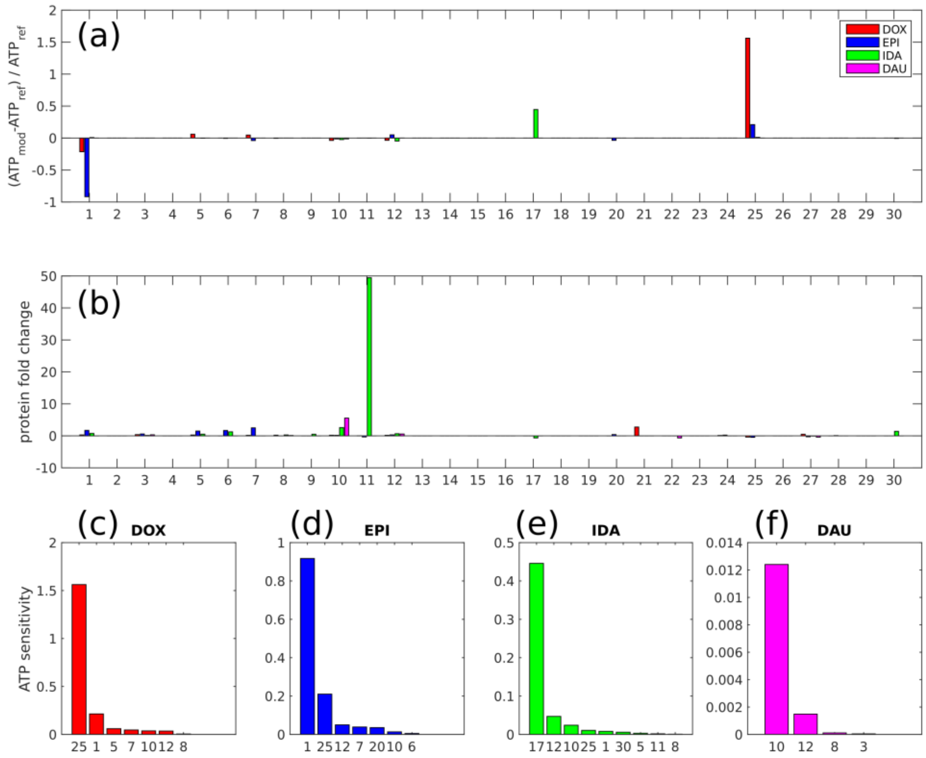

Supplementary Figure 18 Sensitivity analysis of mitochondrial model. (a) Simulated relative change in ATP concentration following a macroscopic change in the drug-induced (toxic dose) protein change after 7-day exposure. (b) Corresponding protein fold change. (c-f) Absolute values of the ATP sensitivities, sorted according to magnitude for doxorubicin, epirubicin, idarubicin, and daunorubicin. The numbers on the horizontal axes refer to the reactions labelled in Fig. 6a and Supplementary Fig. 19.

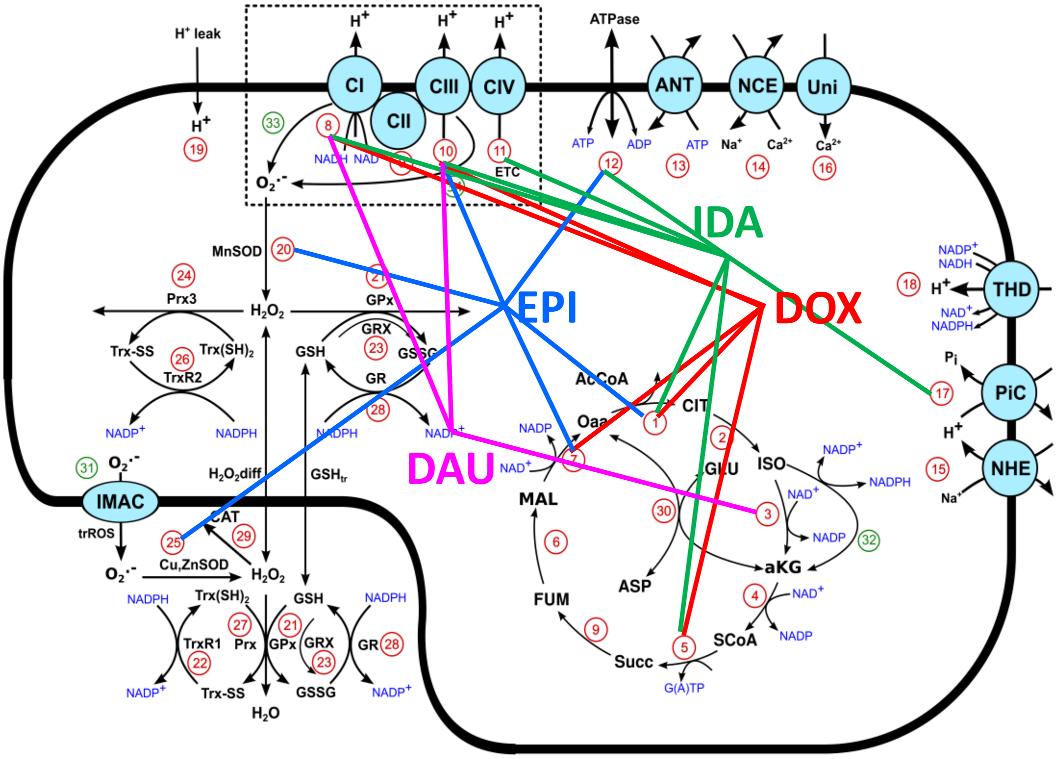

Supplementary Figure 19 ACT response network proteins cover model components that mostly influence ATP concentration. Mitochondrion reaction system with labelled transitions. Colored lines indicate model components that have been identified by network modules computed from integrated (transcriptome and proteome) *in vitro* microtissue data and that have significant impact on ATP concentration as computed by model sensitivity analysis in Supplementary Fig. 18.

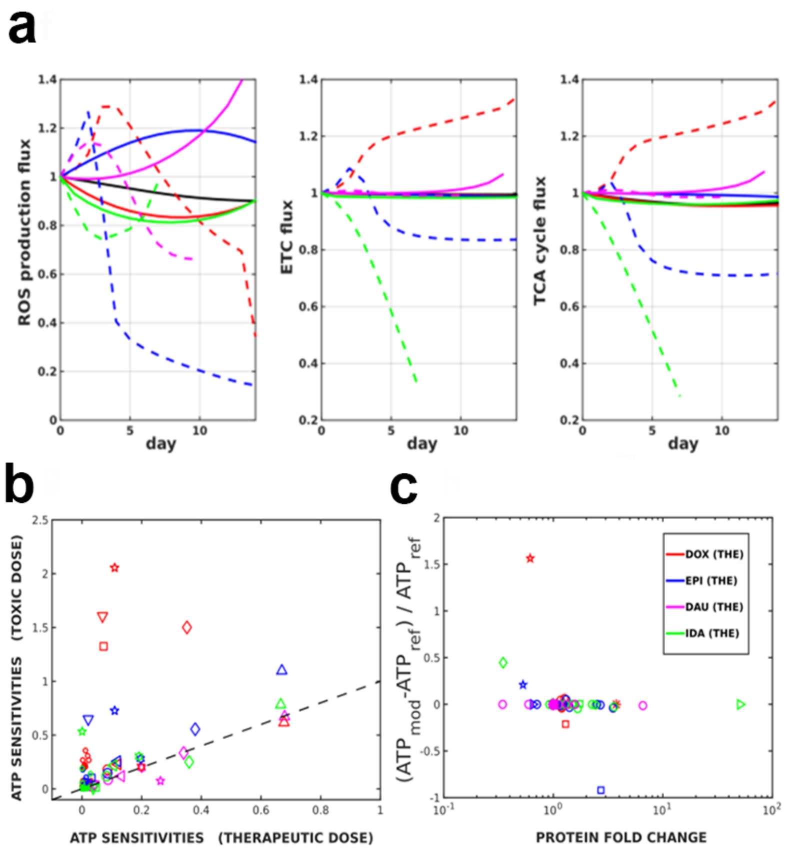

Supplementary Figure 20. (**a**) Simulated fluxes through the subsystems at steady state, as functions of time over the course of drug exposure. (ETC flux: mean flux through complexes CI-CIV; TCA flux: mean flux through the TCA cycle transitions; ROS flux: total flux of superoxide production given by the sum of the fluxes from ETC Complexes I and III.) (**b**) Comparison of sensitivities of ATP concentration with respect to each reaction in the model, following exposure to either the toxic dose (vertical axis) or the therapeutic dose (horizontal axis). Sensitivities were calculated by introducing a 1% change to the reaction activities. The dashed line has unit gradient. Symbols refer to the reactions marked in Figure 6a. (**c**) Sensitivity of ATP concentration to macroscopic changes in reaction activity (see Methods), plotted as a function of the fold change in protein density. Symbols refer to the reactions marked in Fig. 6a and Supplementary Fig. 19.

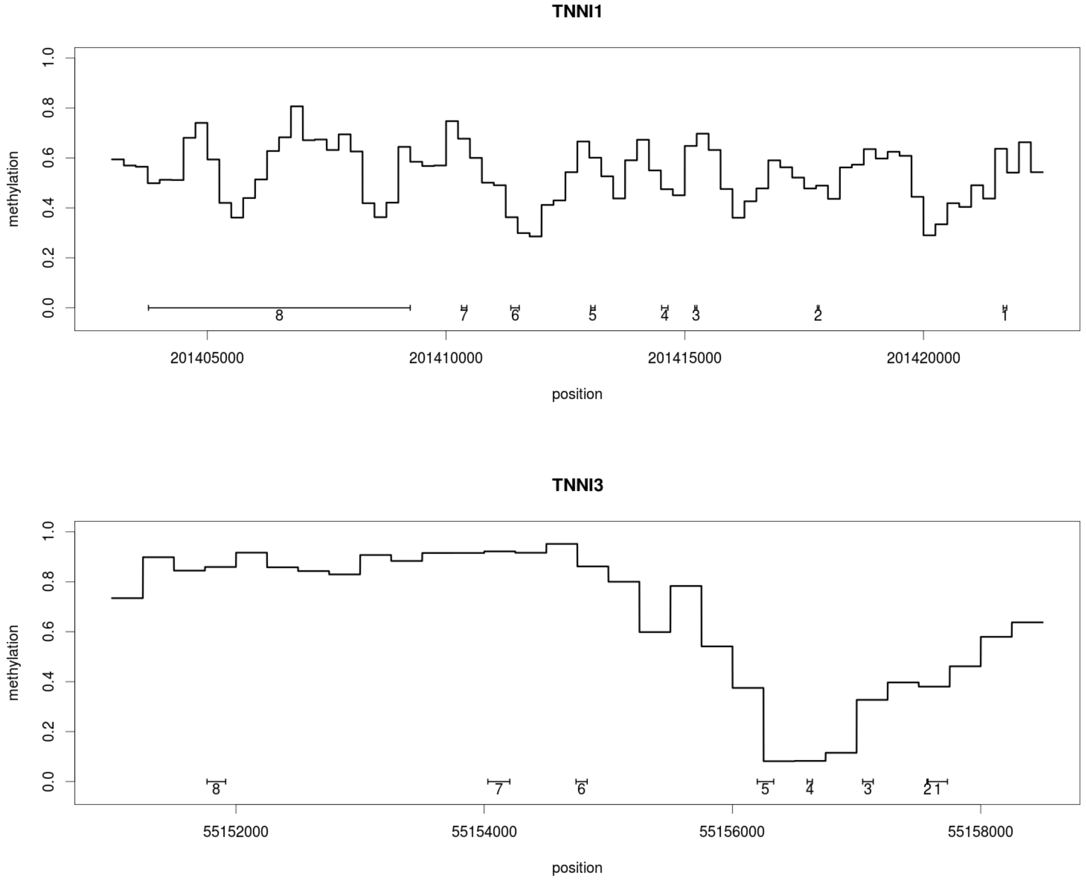

Supplementary Figure 21. TNNI1/TNNI3 gene body methylation in control microtissues.

Supplementary Information References

[1] Conesa, A., Nueda, M.J., Ferrer, A., Talon, M. maSigPro: a method to identify significantly differential expression profiles in time-course microarray experiments. *Bioinformatics* **22**, 1096-1102 (2006).

[2] Lienhard, M., et al., QSEA-modelling of genome-wide DNA methylation from sequencing enrichment experiments. *Nucleic Acids Res.* **45**, e44 (2017).

[3] Herwig, R., Hardt, C., Lienhard, M., Kamburov, A. Analyzing and interpreting genome data at the network level with ConsensusPathDB. *Nat. Protoc.* **11**, 1889-1907 (2016).

[4] Barel, G., Herwig, R. Network and pathway analysis of toxicogenomics data. *Front. Genet.* **9**, 484 (2018).

[5] Leiserson, M.D., et al. Pan-cancer network analysis identifies combinations of rare somatic mutations across pathways and protein complexes. *Nat. Genet.* **47,** 106-114 (2015).

[6] Ziller, M.J. et al. Charting a dynamic DNA methylation landscape of the human genome. *Nature* **500**, 477-481 (2013).

[7] Verheijen, M. et al. Bringing in vitro analysis closer to in vivo: Studying doxorubicin toxicity and associated mechanisms in 3D human microtissues with PBKK-based dose modelling. *Toxicol. Lett.* **294**, 184-192 (2018).

[8] Talay, U., Prasad, S. & Cook, S.A. Genetics and genomics of dilated cardiomyopathy and systolic heart failure. *Genome Med.* **9**, 20 (2017).
