# Supplementary material for: Network integration and modelling of dynamic drug responses at multi-omics levels
Source: Commun Biol. 2020 Oct 15;3:573. doi: 10.1038/s42003-020-01302-8 (PMC7567116; doi:10.1038/s42003-020-01302-8)
Supplement: Supplementary file 2 — Description of Additional Supplementary Files [file 42003_2020_1302_MOESM2_ESM.docx]

**Description of Additional Supplementary Files**

**File Name: Supplementary Data 1**

**Description:** 2 worksheets.

“Promoter and Gene Body DMRs”: List of genes with DMRs in promoters and gene bodies common with all four anthracycline treatments at therapeutic and toxic doses.

“ORA-DMRs”: Over-representation analysis of genes with promoter and gene body DMRs (all anthracyclines) with respect to human pathways (Q<0.05).

**File Name: Supplementary Data 2**

**Description:** 3 worksheets.

“Dynamic response proteins”: Proteins identified with LC-MS that were found significantly altered between anthracycline treatment and DMSO control with temporal polynomial regression model

“ORA-GOs”: Over-representation analysis of dynamic response proteins with gene ontology (GO) terms (Q<0.01)

“ORA-Pathways”: Over-representation analysis of dynamic response proteins with pathways (Q<0.01)

**File Name: Supplementary Data 3**

**Description:** 3 worksheets.

“Dynamic response genes”: Genes measured with RNA-seq that were found significantly altered between anthracycline treatment and DMSO control with temporal polynomial regression model

“ORA-GOs”: Over-representation analysis of dynamic response genes with gene ontology (GO) terms (Q<0.01)

“ORA-Pathways”: Over-representation analysis of dynamic response genes with pathways (Q<0.01)

**File Name: Supplementary Data 4**

**Description:** 3 worksheets.

“Module genes – Proteome data”: Lists of genes identified with network propagation based on proteome data for all four anthracycline treatments at two doses.

“Module genes – Transcriptome data”: Lists of genes identified with network propagation based on transcriptome data for all four anthracycline treatments at two doses.

“Module genes – Integrated data”: Lists of genes identified with network propagation based on combined proteome and transcriptome data for all four anthracycline treatments at two doses.

**File Name: Supplementary Data 5**

**Description:** 2 worksheets

“Patient data”: Patient data for chronic cardiotoxic and control cardiomyopathic patient groups

“Protein expression in biopsies”: Normalized LC-MS values for proteins related to Fig. 5.
