# Supplementary material for: Network integration and modelling of dynamic drug responses at multi-omics levels
Source: Commun Biol. 2020 Oct 15;3:573. doi: 10.1038/s42003-020-01302-8 (PMC7567116; doi:10.1038/s42003-020-01302-8)
Supplement: Supplementary file 8 — Reporting Summary [file 42003_2020_1302_MOESM8_ESM.pdf]

nature research

Corresponding author(s): Ralf Herwig
Last updated by author(s): Sep 8, 2020

Reporting Summary

Nature Research wishes to improve the reproducibility of the work that we publish. This form provides structure for consistency and transparency in reporting. For further information on Nature Research policies, see our Editorial Policies and the Editorial Policy Checklist.

Statistics

For all statistical analyses, confirm that the following items are present in the figure legend, table legend, main text, or Methods section.

n/a	Confirmed	
☐	☒	The exact sample size (n) for each experimental group/condition, given as a discrete number and unit of measurement
☐	☒	A statement on whether measurements were taken from distinct samples or whether the same sample was measured repeatedly
☐	☒	The statistical test(s) used AND whether they are one- or two-sided<br>*Only common tests should be described solely by name; describe more complex techniques in the Methods section.*
☐	☒	A description of all covariates tested
☐	☒	A description of any assumptions or corrections, such as tests of normality and adjustment for multiple comparisons
☐	☒	A full description of the statistical parameters including central tendency (e.g. means) or other basic estimates (e.g. regression coefficient) AND variation (e.g. standard deviation) or associated estimates of uncertainty (e.g. confidence intervals)
☒	☐	For null hypothesis testing, the test statistic (e.g. F, t, r) with confidence intervals, effect sizes, degrees of freedom and P value noted<br>*Give P values as exact values whenever suitable.*
☒	☐	For Bayesian analysis, information on the choice of priors and Markov chain Monte Carlo settings
☒	☐	For hierarchical and complex designs, identification of the appropriate level for tests and full reporting of outcomes
☒	☐	Estimates of effect sizes (e.g. Cohen's d, Pearson's r), indicating how they were calculated

Our web collection on statistics for biologists contains articles on many of the points above.

Software and code

Policy information about availability of computer code

Data collection	Alignment of methylation sequencing data to the reference genome was done with bwa (Version 0.7.15-r1140), further analysis steps were done with the R/Bioconductor package QSEA (http://bioconductor.org/packages/release/bioc/html/qsea.html). The code for collecting the proteomics and transcriptomics data is proprietary (Genedata Expressionist® and Profiler® software v.11.0).
Data analysis	The code for analyzing the methylome data is available through the R/Bioconductor package QSEA (http://bioconductor.org/packages/release/bioc/html/qsea.html), and the code for performing the longitudinal proteome and transcriptome data analysis through the R/Bioconductor package masigpro (https://www.bioconductor.org/packages/release/bioc/html/maSigPro.html). The code for processing and normalizing the proteomics and transcriptomics data is proprietary (Genedata Expressionist® and Profiler® software v.11.0). The code for performing the network propagation is available from github (https://github.com/raphael-group/hotnet2).

For manuscripts utilizing custom algorithms or software that are central to the research but not yet described in published literature, software must be made available to editors and reviewers. We strongly encourage code deposition in a community repository (e.g. GitHub). See the Nature Research guidelines for submitting code & software for further information.

Data

Policy information about availability of data

All manuscripts must include a data availability statement. This statement should provide the following information, where applicable:

- Accession codes, unique identifiers, or web links for publicly available datasets
- A list of figures that have associated raw data
- A description of any restrictions on data availability

Data have been submitted to the BioStudies repository (https://www.ebi.ac.uk/biostudies/) and is available under the following accession numbers:
- Methylation data: S-HECA339, S-HECA340, S-HECA341, S-HECA343, S-HECA347, S-HECA352, S-HECA353, S-HECA363, S-HECA431, S-HECA432, S-HECA433, S-

HECA434.
- Proteomics data: S-HECA2, S-HECA3, S-HECA19, S-HECA20, S-HECA21, S-HECA22, S-HECA38, S-HECA39, S-HECA54.
- RNA-seq data: S-HECA10, S-HECA11, S-HECA12, S-HECA148, S-HECA151.

Field-specific reporting

Please select the one below that is the best fit for your research. If you are not sure, read the appropriate sections before making your selection.

- [x] Life sciences
- [ ] Behavioural & social sciences
- [ ] Ecological, evolutionary & environmental sciences

For a reference copy of the document with all sections, see nature.com/documents/nr-reporting-summary-flat.pdf

Life sciences study design

All studies must disclose on these points even when the disclosure is negative.

Sample size

Longitudinal data analysis: This analysis was performed with the transcriptome and proteome data with the goal to identify response genes/proteins that have an expression profile over time that is different from the control experiments. This analysis was done using the R/Bioconductor package MaSigPro and applying a two-step polynomial regression model with maximal degree of 2. For each treatment and dose the respective 21 experiments (7 time points x 3 replicates) along with the 21 control experiments were summarized into the polynomial model and significant deviations were identified according to the respective P-values.

Time-point specific data analysis: This analysis was performed with the transcriptome and proteome data with the goal to identify differentially expressed genes/proteins at single time points comparing the replicates per time point (3x treatment vs 3x controls) with a statistical test (in the case of transcriptome data with DESeq2, in the case of proteome data with Student's t-test).

Pooled time point analysis: This analysis was performed with the methylation data in order to identify differentially methylated regions (DMRs) between AC treatment and controls across the seven time points, with prior averaging of the replicates per time point, using the QSEA tool (7x treatment vs 7x controls). QSEA transforms the MeDIP-seq enrichment counts for each genomic region into a bisulfite-like % methylation value using a Bayesian model. The statistical analysis for identifying DMRs is then based on a generalized linear model.

Data exclusions

Methylome analysis
QC was performed based on the number of paired-end reads mapped to the reference genome, the coverage of the genome sequence and follow-up visual inspection of all experiments per treatment group using PCA. This excluded 2 out of 130 (1.5%) experiments.

Transcriptome analysis
First, experiments with an insufficient number of mapped paired reads were discarded. Additionally, we applied visual inspection using PCA, heatmaps based on expressed genes and Cook's distance measures. Where these measures gave consistent negative results we flagged the experiments as 'outliers' and excluded them from further analyses. In total 5 out of 186 (2.7%) experiments were discarded.

Proteome analysis
QC was performed on visual inspection using PCA. This excluded 6 out of 186 (3.2%) experiments.

Replication

Each experiment was performed in 3 replicates and replication was judged by correlation analysis.

Randomization

No randomization. Covariates (replicates, time points etc.) were introduced in the polynomial (transcriptome, proteome) and generalized linear (methylome) models.

Blinding

Blinding was not relevant for this study, because we did not develop a prediction method.

Reporting for specific materials, systems and methods

We require information from authors about some types of materials, experimental systems and methods used in many studies. Here, indicate whether each material, system or method listed is relevant to your study. If you are not sure if a list item applies to your research, read the appropriate section before selecting a response.

Materials & experimental systems

n/a	Involved in the study	
x		Antibodies
	x	Eukaryotic cell lines
x		Palaeontology and archaeology
x		Animals and other organisms
	x	Human research participants
x		Clinical data
x		Dual use research of concern

Methods

n/a	Involved in the study	
x		ChIP-seq
x		Flow cytometry
x		MRI-based neuroimaging

Eukaryotic cell lines

Policy information about cell lines

Cell line source(s)	Commercially available human iPSC-derived cardiomyocytes were obtained from Cellular Dynamics International Inc. (CDI, Madison, WI, USA), Catalog N C1056. Human Cardiac Fibroblasts- adult ventricle (HCF-av) were purchased from ScienCell Research Laboratories (San Diego, California, US), Catalog N 6310. The cardiomyocytes and fibroblasts were aggregated into 3D cardiac spheroids by InSphero AG (Schlieren, Switzerland).
Authentication	The authentication of both cell types was performed by commercial provider, CDI and ScienCell, correspondingly.
Mycoplasma contamination	The both cell types were proved to be mycoplasma-negative by the corresponding provider.
Commonly misidentified lines (See ICLAC register)	n/a

Human research participants

Policy information about studies involving human research participants

Population characteristics	Cases with a previous history of cardiotoxic -anthracycline- chemotherapy (n=7) were selected, as well as control DCM/HNDC without (n=8). Endomyocardial biopsies (EMB) were performed as part of routine diagnostic work-up in non-ischemic, non-valvular cardiomyopathy, upon consent of the patient, as part of the Maastricht Cardiomyopathy Registry. 13 patients were female (because of the clinical indications of the cardiotoxic group which is predominantly previous breast cancer therapy), 2 were male. In each group age, gender and BMI were matched.
Recruitment	Patients were selected from the Maastricht Cardiomyopathy Registry.
Ethics oversight	The study was performed according to the declaration of Helsinki and was approved by the Medical Ethics Committee of Maastricht University Medical Centre. All patients gave written informed consent.

Note that full information on the approval of the study protocol must also be provided in the manuscript.
